# Supplementary figures and images for: Participatory mental health interventions in low-income and middle-income countries: a realist review protocol
Source: BMJ Open. 2022 Apr 6;12(4):e057530. doi: 10.1136/bmjopen-2021-057530 (PMC8991062; doi:10.1136/bmjopen-2021-057530)

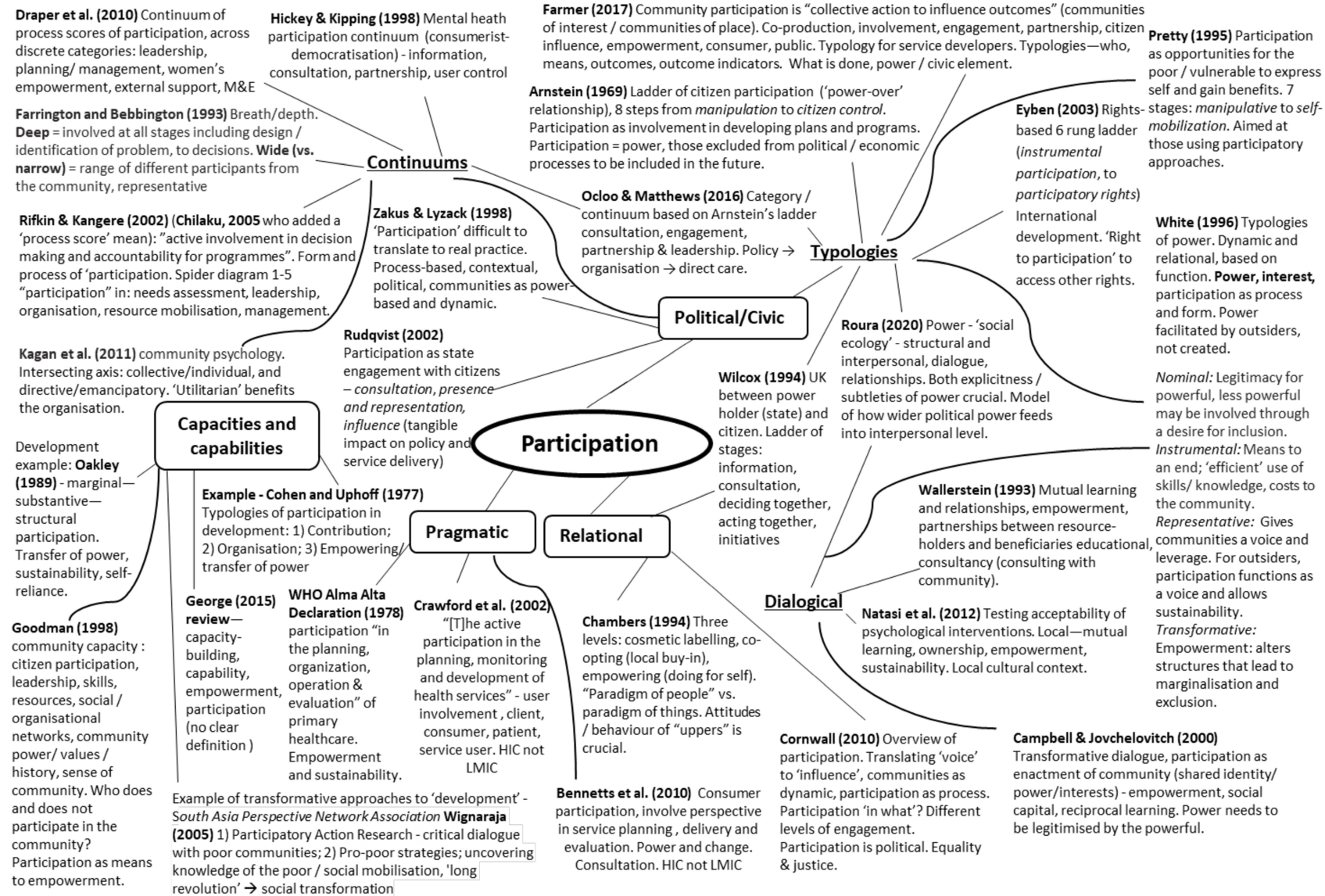

Supplement: Supplementary data [file bmjopen-2021-057530supp001.pdf]
