# Supplementary material for: Participatory mental health interventions in low-income and middle-income countries: a realist review protocol
Source: BMJ Open. 2022 Apr 6;12(4):e057530. doi: 10.1136/bmjopen-2021-057530 (PMC8991062; doi:10.1136/bmjopen-2021-057530)
Supplement: Supplementary data [file bmjopen-2021-057530supp002.pdf]

Search strategy for CIHANL, PsycINFO, WebofScience, ASSIA, Medline, Embase

((participat* OR empower* OR community OR "community?led" OR co-designed OR inclusion OR inclusive OR "capacity building" OR capabilities OR engagement OR consultation OR co-produc* OR peer-led OR peer?to?peer OR task?shifting OR task?switching) **AND** ("Psychological disorder" OR "Psychological problem" OR "Psychological illness" OR "Psychological distress" OR "Psychiatric disorder" OR "Psychiatric problem" OR "Psychiatric illness" OR "Psychiatric distress" OR "Psychosocial disability" OR "Mental illness" OR "Mental health" OR SMI) **AND** ("Developing countr*" OR "low?income countr*" OR "middle?income countr*" OR LMIC OR "third world"))

Limits: Peer reviewed

Limits: Date 2007-2021

Search strategy for JSTOR

[JSTOR which has a 200-character word limit. Discussion was had among the team via email and during monthly team meetings, as to how to truncate the search most effectively]

((participat* OR community OR inclus*) AND ab:(psychological OR "psychiatric disorder*" OR "Psychosocial disability*" OR "Mental illness*" OR "Mental health") AND ("developing countries" OR LMIC))

Limits: Date 2007-2021
