# Supplementary material for: Participatory mental health interventions in low-income and middle-income countries: a realist review protocol
Source: BMJ Open. 2022 Apr 6;12(4):e057530. doi: 10.1136/bmjopen-2021-057530 (PMC8991062; doi:10.1136/bmjopen-2021-057530)
Supplement: Author's manuscript [file bmjopen-2021-057530.draft_revisions.pdf]

BMJ Open

BMJ Open is committed to open peer review. As part of this commitment we make the peer review history of every article we publish publicly available.

When an article is published we post the peer reviewers' comments and the authors' responses online. We also post the versions of the paper that were used during peer review. These are the versions that the peer review comments apply to.

The versions of the paper that follow are the versions that were submitted during the peer review process. They are not the versions of record or the final published versions. They should not be cited or distributed as the published version of this manuscript.

BMJ Open is an open access journal and the full, final, typeset and author-corrected version of record of the manuscript is available on our site with no access controls, subscription charges or pay-per-view fees (http://bmjopen.bmj.com).

If you have any questions on BMJ Open's open peer review process please email info.bmjopen@bmj.com

BMJ Open

Participatory mental health interventions in low and middle income countries: a rapid realist review protocol

Journal:	*BMJ Open*
Manuscript ID	bmjopen-2021-057530
Article Type:	Protocol
Date Submitted by the Author:	20-Sep-2021
Complete List of Authors:	Heap, Cheyann ; University of York, Department of Social Work and Social Policy<br>Jennings, Hannah; University of York; University College London<br>Gaire, Himal; Centre for Mental Health Counselling (CMC)<br>Gumbonzvanda, Farirai; Rozaria Memorial Trust<br>Gumbonzvanda, Nyaradzayi; Rozaria Memorial Trust<br>Gupti, Garima; Burans, Herbertpur Christian hospital<br>Jain, Sumeet; The University of Edinburgh School of Social and Political Science<br>Maharjan, Bidya; Chhahari Nepal for Mental Health (CNMH)<br>Maharjan, Rakchhya; Chhahari Nepal for Mental Health (CNMH)<br>Maharjan, Sujen Man; Chhahari Nepal for Mental Health (CNMH)<br>Mahat, Pashupati; Centre for Mental Health and Counselling<br>Mathias, Kaaren; University of Canterbury, School of Health Sciences; Burans, Herbertpur Christian hospital<br>Pillai, Pooja; Burans, Herbertpur Christian hospital<br>Webber, Martin; University of York, Department of Social Policy and Social Work<br>Wright, Jerome; University of York<br>Burgess, Rochelle; UCL Institute for Global Health
Keywords:	MENTAL HEALTH, PUBLIC HEALTH, QUALITATIVE RESEARCH

SCHOLARONE™
Manuscripts

Participatory mental health interventions in low and middle income countries: a rapid realist review protocol

Authors:

Cheyann J Heap[1]; Hannah Maria Jennings[2 3 4 *]; Himal Gaire[5]; Farirai Gumbonzvanda[6]; Nyaradzayi Gumbonzvanda[6]; Garima Gupti[7]; Sumeet Jain[8]; Bidya Maharjan[9]; Rakchhya Maharjan[9]; Sujen Man Maharjan[9]; Pashupati Mahat[5]; Kaaren Mathias[7 10]; Pooja Pillai[7]; Martin Webber[1]; Jerome Wright[2]; Rochelle A Burgess[4]

[1] Department of Social Work and Social Policy, University of York, UK

[2] Department of Health Sciences, University of York, UK

[3] Hull York Medical School, University of York, UK

[4] Institute for Global Health, University College London, UK

[5] Centre for Mental Health and Counselling (CMC), Nepal

[6] Rozaria Memorial Trust (RMT), Zimbabwe

[7] Burans, Herbertpur Christian hospital, India

[8] School of Social and Political Science, University of Edinburgh, UK

[9] Chhahari Nepal for Mental Health (CNMH), Nepal

[10] School of Health sciences, University of Canterbury, New Zealand

*corresponding author: hannah.jennings@york.ac.uk Department of Health Sciences, University of York, Heslington, York YO10 5DD

Word count: 3,435 words

For peer review only

ABSTRACT

Introduction: The launch of the Movement for Global Mental Health brought longstanding calls for improved mental health interventions in low-and middle-income countries (LMICs) to centre stage. Within the movement, the participation of communities and people with lived experience of mental health problems is argued as essential to successful interventions. However, there remains a lack of conceptual clarity around 'participation' in mental health interventions with the specific elements of participation rarely articulated. Our review respond to this gap by exploring how 'participation' is applied, what it means and what key mechanisms contribute to change in participatory interventions for mental health in LMICs.

Methods and analysis: A rapid realist review methodology will be used to identify the different contexts which trigger mechanisms behind the outcomes of participatory mental health interventions, that is: what makes participation *work* in mental health interventions in LMICs, and *why*? We augment our search with primary data collection in communities who are the targets of global mental health initiatives, to inform the production of a programme theory on participation for mental health in LIMCs.

Ethics and dissemination: Ethical approval for FGDs was sought from ethical bodies in each country involved**.** FGDs will be conducted in line with WHO safety guidance during the covid-19 crisis. The full review will be published in an academic journal, with further papers providing an in-depth analysis on community perspectives on participation. The project findings will also be shared on a website, in webinars and an online workshop.

Keywords: participation, mental health interventions, realist review, LMICs, community, empowerment, involvement

For peer review only

STRENGTHS AND LIMITATIONS

- The review is strengthened by stakeholder involvement - focus groups with people with mental health problems and carers in LMICs.
- The review is theory-driven, using concepts of participation from academic literature and applied practice as a framework.
- The review is limited to published academic papers in English; there may be further insights available in grey literature and in other languages.

For peer review only

INTRODUCTION

Global Mental Health

Globally, mental ill health is a leading cause of disability[1]. Socio-structural factors are increasingly recognised as causes and consequences of poor mental health[2-3]. In 2007, *The Lancet's* pivotal 'Global Mental Health' series highlighted global inequalities in mental health provision, particularly in low-to-middle-income countries (LMICs)[4], marking the beginning of what is now commonly referred to as the *movement for Global Mental Health*[5]. The movement advocates evidence-based interventions, informed by a human rights approach, with the aim to scale up mental health services. However, these efforts operate primarily within an essentially biomedical framework that eschews attention to structural drivers of distress in favour of pragmatics in clinical care. The movement has faced extensive critique because of this approach, with a special section of Transcultural Psychiatry in 2012 arguing for a greater emphasis of engagement with local communities within mental health to overcome these limitations[6]. For example when communities actively participate in the governance, design or delivery of mental health interventions they can be more acceptable and relevant to local needs as well as more cost-effect more and higher quality[7]. Furthermore, depending on the social processes involved, participation can be empowering and even transformative to communities[8-9]. However, an inattention to power dynamics when working on 'participation' run the risk of exacerbating existing patterns of exclusion and reinforcing inequities[8].

As a group of researchers and practitioners, we are interested in the theory and application of participation in global mental health interventions in LMICs for two key reasons: First, we argue that how people in LMICs define participation in mental health interventions or more broadly matters to programmes. Second, we argue that participation in mental health interventions creates platforms for transformation in the lives of people it seeks to benefits *if* attention is paid to key processes.

For peer review only

Theories of participation

Debates around the active involvement of communities in localities in LMICs – referred to as ‘participation’, ‘community engagement’ and ‘mobilisation’ – are important and related principles can be found within the broader fields of global health and international development. The diversity of approaches relating to the term, also lead to its evolution into what some argue as a benign concept, a term with wide reaching boundaries which enable its wide ranging applications[10]. There are different perspectives on how ‘participation’ is achieved and for what purposes participation is implemented[9]. For example, Campbell and Jovchelovitch suggest that participation is a mechanism for negotiating social identity, and participatory power thus emerges as “a space of possible action”[11]. Others suggest that mental health ‘consumer participation’ must include the power to take meaningful action through orchestrating changes in service planning, delivery and intervention[12-13]. What action looks like is dependant on the specific theoretical approach applied in a certain context.

Participatory theories can be broadly separated into *typologies* (categories, often set as ‘stages’) or *continuums* (spectrums). ‘Ladder’ or stage typologies are common in civic, political understandings of power. Arnstein’s famous (1969) ladder of citizen participation has eight rungs from *manipulation* to *citizen control*[14]. Similarly, Goetz and Gaventa range from *consultation* (information sharing) to *influence* (tangible impact on policy making and service delivery)[15], and Wilcox considers collaboration between citizen and state from *information* to *initiatives*[16]. Pretty frames ‘participation’ as opportunities for the poor to gain benefits, from *manipulative* to *self-mobilization*[17], whereas Eyben’s rights-based, six-rung ladder (*instrumental participation* to *participatory rights*) suggests that the right to participation facilitates access to other human rights[18]. These ladders/stages have several commonalities: on lower rungs people have little power, and any inclusion of their voices is tokenistic and does not lead to tangible change. The top rungs are interventions designed and led by the community. However, ladders do not effectively accommodate the dynamic, contextual and relational

For peer review only

aspects of power in participation. Additionally, a fully citizen-led approach may not always be possible or even desirable.

Continuums can accommodate shifts in participation and power status. In community development, Draper et al. created a process-based continuum of participation across five categories including external support, leadership and women's empowerment[19]. Bebbington and Farrington's continuum focuses on participation breadth (representativeness of the full community), and depth (extent of meaningful involvement)[20]. Similar to participation ladders, Hickey and Kipping's mental health participation continuum ranges from *information* to *user control*, with a shift from passive 'consumerism' to 'democratisation'[21]. Likewise, Rifkin and Kangere argue participation ranges from people as passive receivers to active decision makers, acknowledging change over time [22].

Some approaches emphasise the dialogical nature of participation. Campbell and Jovchelovitch take a Freirean approach, noting how transformative dialogue can lead to reciprocal growth and learning between 'the powerful' and 'the community'[23]. Wallerstein also highlights the capacity for mutual learning and reciprocal relationships, with partnerships between resource-holders and beneficiaries leading to transformational change for both[24]. The South Asia Perspective Network Association goes further to emphasise deliberately 'pro-poor' strategies, where through a dialogic approach, sustained participatory action and new social movements lead to wider social transformation[25]. Nastasi et al.'s Participatory Intervention Model applies this idea to mental health interventions, noting the importance of relationships, changing interpersonal and power dynamics, and matching knowledge and support to the needs of the community[26]. Indeed, communities are constantly shifting and have their own internal power dynamics[27]. White takes account of this in a relationally-based typological approach, which accounts for shifting priorities and dynamics between the community and those with power[28]. This typology ranges from *nominal* (tokenistic involvement), to *transformative* (aiming to empowerment).

For peer review only

Supplemental file 1 shows our mind map bringing together key debates in participation based around who it is for, how it is used, and the impact (if any) on participatory approaches. The mind map is intended to be comprehensive but not exhaustive. It was supplemented by discussion on the lived experiences of practitioners in the team and the participatory work of their organisations[29-30]. We conceptualise 'participation' in mental health as *the active involvement of people affected by interventions or targeted action.* This can include a range of actors: service users, carers, providers, and wider community members. Ultimately, they all become agents who contribute to the intervention in some capacity, and are not merely passive recipients of an outcome. This includes involvement and ownership over various phases of concept, design, implementation, and evaluation stages of interventions. Notably, our approach recognises a range of dynamics and interest which reflect on how the following factors shape participation including: interests and motivations, socio-economic context, influence, and structural and interpersonal power.

Rapid Realist Review on Participation on Mental Health in LMIC

To date there have been reviews of community engagement and participatory health approaches in LMICs[31-32] including an ongoing realist review on community engagement in non-communicable disease interventions and research[33], however to the authors' knowledge there has been no specific review which interrogates participatory approaches to mental health across LMICs. The current review seeks to address this gap exploring the following questions: *what do participatory approaches look like, how they are used, and what (if anything) is their impact?*

Our investigation applies a realist methodology, which has a similar interest in "what works for these particular people in particular contexts"[31,34]. Realists review combine theory and empirical observation and seek to understand how and why change is facilitated[31,33,34, 35]. A rapid realist review (RRR) can therefore explore phenomena though synthesising and comparing information within a

For peer review only

limited timeframe. This review will be used to identify mechanisms of change and meaningfully develop a theory despite interventions themselves being context-dependent and heterogeneous. Using our mind map as a starting point our review will be used to develop and refine a programme theory of 'what works' in these types of interventions, to guide future projects carried out by our network of researchers and practitioners.

Aim and research questions

The aim of the review is to map existing literature in participatory mental health interventions in low- to middle-income countries and consider how participatory approaches work. There will be two stages of data extraction. We will answer the following questions through the review:

1. Why and for whom has participation been used in mental health interventions in LMIC?
2. How has participation been operationalised in this research?
3. To what extent, based on existing participation frameworks?
4. What are the mechanisms of action of participation in this context?
5. What are the effects/outcomes of participation on the mental health of individuals and communities?

METHOD

This review was designed adapting Saul et al's suggested 10 steps for a realist review[36]. Realist reviews often involve developing and refining middle-range theories of what works[36-38]. Middle-range theories are general enough to be transferred to other projects, but concrete and specific enough to apply to practice[34]. In generating or exploring a theory Context, Mechanism and Outcomes are crucial, these are known as CMO configurations **[Figure 1: CMO configurations]**. CMO configurations are guided by the research question[38]. Here, we are asking about mechanisms of action related to the extent to

For peer review only

which participation happens, and how 'participation' happens in research. We are also asking what effect, if any, participation has on mental health (with the corresponding mechanisms of action). CMO configurations are iteratively extracted and developed during data extraction and synthesis.

Figure 1: CMO configurations

As not all papers will have enough detail to infer theories and extract CMO relationships, data extraction will be in two stages. The first will be surface-level study characteristics, and quality. The second stage will be a deeper analysis of the highest quality papers, from which CMO configurations will be extracted and overall middle-range theories created. To counter the western hegemonic knowledge structures that shape much of the peer reviewed literature[39] we have added specific stakeholder consultations in our review, to combine and include embodied knowledges held by people with mental health difficulties, carers and community members. We assess their own views of participation and involvement as it relates to mental health, at two stages in the review (see step 7). The 10 steps as we have applied them in our study are described in more detail below **[Figure 2: 10 step review process]**

Figure 2: 10 step review process

Concepts of 'participation' and developing research questions (steps 1 and 2)

A mind map of different concepts of 'participation' **[Supplementary file 1]** was created between January and March 2021. This involved reading research literature on participation, particularly on LMICs and from the academic fields of development, health and human rights. This was supplemented by monthly meetings between the entire research group, to discuss emerging themes and link the research to the applied practice of team members. Team members, across LMICs and high-income countries, represent varied ethnic backgrounds and disciplinary backgrounds in mental health, medicine, social work, mental health nursing, psychology, academia, and non-governmental

For peer review only

organisations. Key debates from the mindmap and group discussion were used to develop the five research questions.

Based on this initial conceptual review and discussions, project PI (RB) drafted an initial programme theory and model which was discussed and refined within the research group **[Figure 3: programme theory]**. The programme theory guided the remainder of the review process and will be refined in light of the findings of this review (see step 9). The programme theory states that individuals who live with or are at risk of mental health conditions in the global south (LMIC) because of a number of day to day realities across various settings with relations to mental health needs (contexts) which include: poor mental health service infrastructure; low uptake of services due to low awareness of services and due to a poor 'fit' of services to the needs of communities; stigma and exclusion faced by people living with mental health issues; histories of silencing of lived experience within the mental health space; and intersecting social, political, and environmental challenges that link to mental health conditions. *If* we have participatory approaches to mental health that meet the parameters of *meaningful participation* (as defined in our conceptual review) there would be a positive impact on individual mental health and wider societal issues linked to poor mental health. The potential outcomes would include: improved mental health outcomes; improved access to services, improved mental health literacy, a reduction in experiences of exclusion, a reduction in harmful social, political and environmental drivers of distress (i.e. reduced poverty; reduced gender discrimination; improved living conditions) and increased voice and ownership for people with lived experience in wider society.

Figure 3: Programme theory

Search terms and identifying papers (steps 3 and 4)

Search terms and databases searched were discussed in team meetings in early 2021. Searches are being carried out in the databases: Scopus, Medline/PsychINFO, ASSIA, CINAHL, EMBASE and JSTOR.

For peer review only - http://bmjopen.bmj.com/site/about/guidelines.xhtml

If additional studies are identified in the references or through the team they will be considered for screening. **Table 1** shows our search terms.

Table 1: Literature search terms

<table>
<tr><td colspan="3">The overall search formula included three umbrella concepts: ((‘Participation’) AND (‘Mental health’) AND (‘LMICs’)). The terms under each umbrella (e.g. ‘Participation’) were separated by the Boolean operator OR**</td></tr>
<tr><th>‘Participation’</th><th>‘Mental Health’**</th><th>‘LMICs’ (Low to Middle Income Countries)</th></tr>
<tr><td>• participat*
• empower*
• community
• community?led
• co-designed
• inclusion
• inclusive
• capacity building
• capabilities
• engagement
• consultation
• co-produc*
• peer-led
• peer?to?peer
• task?shifting
• task?switching</td><td>• psychological disorder
• psychological problem
• Psychological illness
• Psychological distress
• psychiatric disorder
• psychiatric problem
• psychiatric illness
• psychiatric distress
• psychosocial disability
• mental illness
• mental health
• SMI [Serious Mental Illness]</td><td>• Developing countr*
• low?income countr*
• middle?income countr*</td></tr>
<tr><td colspan="3">**phrases of more than one word were put into “double quotes” for the search
***There are a wide range of conceptions of mental health, including medical (“mental illness”) and psychosocial[40]. We tried to accommodate this variety in our search terms.</td></tr>
</table>

Screening and study selection

Instead of limiting to a particular definition of ‘participation’, this review will take a pragmatic approach. It will include all papers which either: a) claim to be participatory, or b) clearly include participation (the active involvement of people with mental health problems and their supporters). We will include studies located in low- and middle- income countries (as according the World Bank definition) that are related to a mental health intervention. We use a broad definition of mental health

as defined by the WHO and draw on their list of mental and neurological disorders[40]. We define interventions as programmes or policies that are implemented with the aim to change outcome/s. **Table two** shows our inclusion and exclusion criteria.

Table 2: Inclusion and exclusion criteria

Inclusion Criteria	Exclusion criteria
Located in low- and middle- income countries;<br><br>Health and development projects/interventions addressing mental health;<br><br>Intervention, or research study about an intervention, which claims to be participatory OR clearly demonstrates the active involvement of the target population in concept, design, implementation, or evaluation;<br><br>Any 'level' of intervention including individual, group, and systemic interventions such as national and international projects;<br><br>Any method - qualitative, qualitative, or case study;<br><br>Participants are people with mental health problems, their unpaid carers, or other people from the community such as local health workers or laypeople;<br><br>On or after 2001, based on the World Health Report which presented community-based mental health services as critical[41]	'Participation' does not include the active involvement of people from the target community (e.g. laypeople, local professionals, people with mental health problems, carers);<br><br>Not about an intervention ('intervention' = support group, a specific mental health policy, training)

Possible relevant papers identified during the searches will be uploaded onto the software Ryaan for title and abstract screening. Title and abstract screening will be conducted blinded by members of the research team, to ensure consistency 10% of studies screened by two members of the research team. Full text screening will be completed by the whole research team with members working in pairs and

For peer review only

the papers divided among the team. Any uncertainties throughout the process will be discussed within the team at regular team meetings.

Data extraction and quality appraisal (steps 5 and 6)

There will be two stages of data extraction. The first stage will answer the first three research questions of the review.:

1. Why and for whom has participation been used in mental health interventions in LMIC?
2. How has participation been operationalised in this research?
3. To what extent does participation happen, based on existing participation frameworks?

At the end of the first stage, the highest-quality papers with the most detail about theories of participation and context, mechanisms and actions will be identified and taken forward to the second stage. This more in-depth analysis and CMO extraction will answer the final two questions:

4. What are the mechanisms of action of participation in this context?
5. What are the effects/outcomes of participation on the mental health of individuals and communities?

Stage one

As with the full text screening data extraction will be completed in pairs by the research team. A data extraction tool will be developed, and refined as relevant data are extracted. Extracted data will likely include: study aims, methods, why participation is used; concept and theory of 'participation'; target population and stakeholders; how participation is 'done'; outcomes of intervention; level of participation; analysis of power. For quality, a rapid realist review asks whether papers are *relevant* (to the research question) and *rigorous* (of good-enough quality to make a meaningful contribution)[34]. The data extraction tool will include whether papers are 'low, medium and high' relevance. For

For peer review only

'rigour', as we are including all methods (case studies, qualitative and quantitative), specific quality checklists will be selected based on the nature of the papers, primarily drawing from the Joanna Briggs Institute Critical Appraisal tools[42]. Uncertainties as to the data extraction, relevance and rigour will be discussed in the team meetings and Research Associate (CH) and principle investigators (RB and HJ) will do final quality checks.

Stage two

Papers of 'high quality' – rigorous, relevant, and with sufficient information to extract CMO relationships - will be taken forward for the second stage of review. Potential studies for this stage of data extraction will be identified by the research team during stage one. All studies identified and those marked as 'high' for relevance will be considered. Three members of the research team (CH, RB and HJ) will read the shortlisted paper and agree through consensus whether they should go through to the next round of the review. They will record and report back to the wider team the reasoning for the decisions. The criteria for studies to go through to the next round of review include: information and discussion about participation, enough detail to extract information on CMO relationships.

Focus group discussions (step 7)

There will be two rounds of stakeholder consultations through focus group discussions, across four sites in three countries (Nepal, India and Zimbabwe), organised by members of the network who work in participation and mental health (BM, RM, SMM ,PM, PP, NG, FG, GG). The groups will include people affected by mental health difficulties, carers and community members.

The focus group discussions (FGDs) will take place between Stage 1 and 2 of data extraction. The first round of FGDs will explore understandings of participation. In the second round of FGDs a summary of data so far will be presented in order to get feedback. Findings of the FGDs will be compared and

For peer review only

integrated with findings from the data extraction, to help theorise ‘what works and how’, in the way that is considered most participatory in the context. Findings from the FGDs will be reported in our final write-up, dissemination and revision of our programme theory (steps 9 and 10).

Data synthesis and CMO configurations (step 8 and 9)

Stage one

The first stage of data synthesis based on the first round of data collection will be narrative, based around the first three research questions. It will report on the breadth and scope of participation as utilised in mental health settings in LMICs. Comparisons will be made with the FGDs and their understanding of participation.

Stage two

The second round of data synthesis is theory-focused and iterative process based on the ‘high quality papers’. Data synthesis will take the following process:

- Taking our ‘high quality’ papers we will extract 1-4 CMO configurations related to participation from each paper. This will be an iterative process within the network.
- CMO configurations will be combined and refined to identify ‘enabling contexts’, ‘enabling mechanisms’ and possible outcomes (mid-range theories). Again this will be an iterative process involving the whole network.
- Based on these findings and the FGD analysis we will revise our programme theory

We plan to undertake this process as a team, involving workshops lead by KM (who has experience in realist methodology[43]. One workshop will develop our initial CMO configurations based on the ‘high quality papers’. Subsequent team meetings will allow refining of CMO configurations, comparing them against a thematic analysis of FGDs. As theories and CMO configurations are gradually refined, the

For peer review only

process will be carefully documented to justify changes and ensure a rigorous and transparent process. Overall, the synthesis should be such that the weaknesses (or omissions) in one particular paper from the review are accommodated for by the strengths of other papers. Once the review has been completed, we will have a set of middle-range theories, informed by the embodied knowledge of everyday citizens who are often the targets of mental health interventions in LMICS. The theories explain how participatory mental health interventions create changes in local context (C), to trigger socio-psychological mechanisms (M) that lead to new behaviour outcomes (O) in communities of interest. Final CMO configurations will be used to refine the programme theory by RB and HJ which will be feedback and refined by the wider group.

Dissemination and workshop (step 10)

It is anticipated that the current rapid realist review will create a strong theoretical and practice-based foundation for future work in participatory research and practice for mental health in LMIC settings, and other environments of adversity. It will also directly shape the efforts of our network for participatory mental health in LMICs. We will disseminate our findings within a workshop series, this will allow us to expand our network by welcoming a range of interested stakeholders including people with mental health problems, paid and unpaid carers, practitioners, academics and policymakers. We also plan to disseminate our findings though peer review papers, and blogs written in accessible and local languages in each of our country settings.

Patient and public involvement

As part of the review we will have planned stakeholder consultations with people with mental health difficulties, carers and community members in three LMICs (Nepal, India, Zimbabwe). The consultations are an important part of the review and will contribute to our refined programme

For peer review only

theory. As part of our dissemination we will organise workshops with people with mental health difficulties, carers, practitioners and community members in LMICs.

ETHICS AND DISSIMINATION

Ethical approval for the study received from the University of York Health Science and Research Governance committee (HSRGC/2021/438/F) and we have received ethical approval from the relevant bodies of the countries involved (Nepal, India, Zimbabwe). The review has been registered with PROSPERO (ID number CRD42021241787) . The full review is intended to be published in an academic journal, with further papers providing an in-depth analysis on community perspectives on participation. The project findings will also be shared on a website, in webinars and an online workshop.

AUTHORS CONTRIBUTIONS

RB and HJ initiated the review. All members of the network contributed toward the mind-map and conception of the protocol. Following discussions with the whole team CH designed and drafted the initial protocol, which was subsequently reviewed by BM, KM, PM, MW, RM, SM, JW, HJ and RB. BM, PM, KM, GG, PP, FG, NG and CH have taken a lead in planning the FGDs. HJ, RB, BM, PM, KM and PP have organised ethical approval in respective sites. RB and HJ finalised the protocol. All authors approved the final manuscript (CH, HJ, HG, FG, NG, GG, SJ, BM, RM, SM, PM, KM, PP, WM, JW, RB),.

COMPETING INTERESTS STATEMENT

There are no competing interests.

For peer review only

FUNDING STATEMENT

This work was supported by Global Challenges Research Fund Pump Priming Award funded through the UKRI-MRC (2019-2020/2020-21 H0027804)

REFERENCES

1. Patel VP, Saxena S, Lund C. et al. (2018).The *Lancet* Commission on mental health and sustainable development. *The Lancet Commissions* 2018; 392(10157): 1553-1598.
2. Johnson S. Social interventions in mental health: a call to action. *Soc Psychiatry Psychiatr Epidemiol* 2017;52: 245-247.
3. Burgess R, Jain S, Petersen I. et al. Social interventions: a new era for global mental health? *Lancet Psychiatry* 2020;7:118-9.
4. Horton R. Launching a new movement for mental health. *Lancet* 2007;370:806.
5. Cooper S. Global mental health and its critics: moving beyond the impasse. *Crit Public Health* 2016;4:355-8.
6. Campbell C, Burgess R. The role of communities in advancing the goals of the Movement for Global Mental Health. *Transcult Psychiatry* 2012;49:379-95.
7. Ottmann G, Laragy C, Allen J, Feldman P. Coproduction in Practice: Participatory Action Research to Develop a Model of Community Aged Care. *Systemic Practice and Action Research* 2011;24(5):413-27.
8. George AS, Mehra V, Scott K. et al. Community participation in health systems research: a systematic review assessing the state of research, the nature of interventions involved and the features of engagement with communities. *PLoS One* 2015;10:p.e0141091.
9. Farmer J, Taylor J, Stewart E. et al. Citizen participation in health services co-production: a roadmap for navigating participation types and outcomes. *Aust J Prim Health* 2018;23:509-15.
10. Cornwall, A.. Unpacking 'Participation': models, meanings and practices. *Community Development Journal* 2008;*43*(3), 269-283.
11. Campbell C, Jovchelovitch S. Health, community and development: towards a social psychology of participation. *J Community Appl Soc Psychol* 2000;10:255-70
12. Bennetts W, Cross W, Bloomer M. Understanding consumer participation in mental health: Issues of power and change. *Int J Ment Health Nurs* 2011;20:155-164.
13. Crawford MJ, Rutter D, Manley C. et al. Systematic review of involving patients in the planning and development of health care. *BMJ* 2002;325:1263-8.

For peer review only

14. Arnstein SR. (1969). A ladder of citizen participation. *J Am Inst Plann,* 1969*;*35:216-224.

15. Goetz M, Gaventa J. Bringing citizen voice and client focus into service delivery. Brighton, England: *Institute of Development Studies* 2001.

16. Wilcox D. Community participation and empowerment: putting theory into practice. *RRA Notes* 1994;21:78-82.

17. Pretty J. Participatory learning for sustainable agriculture. World Development 1995;23:1247-63.

18. Eyben R. The rise of rights: rights-based approaches to international development. *IDS Policy Briefing* 2003:17;1-4.

19. Draper AK, Hewitt G, Rifkin S. Chasing the dragon: developing indicators for the assessment of community participation in health programmes. *Soc Sci Med* 2010:71;1102-9.

20. Bebbington A, Farrington J. Governments, NGOs and agricultural development: perspectives on changing inter-organisational relationships. *J Dev Stud* 1993*:*29;199-219.

21. Hickey G, Kipping C. Exploring the concept of user involvement in mental health through a participation continuum. *J Clin Nurs,* 1998:7;83-8.

22. Rifkin SB, Kangere M. What is participation? In: Hartley S (eds.) Community-based rehabilitation (CBR) as a participatory strategy in Africa. Ithaca, NY: Cornell University 2002:37-49.

23. Campbell C, Jovchelovitch S. Health, community and development: Towards a social psychology of participation. *J Community Appl Soc Psychol* 2000:10;255-70.

24. Wallerstein N. Empowerment and health: the theory and practice of community change. *Community Dev J* 1993:28;218-27.

25. Wignaraja P. Pro-poor Growth and Governance in South Asia—Decentralisation and Participatory Development. *The Pakistan Development Review* 2005:44;1159–1171)

26. Nastasi BK, Varjas K, Schensul SL. et al. The Participatory Intervention Model: A framework for conceptualizing and promoting intervention acceptability. *Sch Psychol Q* 2000:15;207-32.

27. Zakus D, Lyzack C. Revisiting community participation. *Health Policy Plan* 1998:13; 1-6.

28. White, S. Depoliticizing development: the uses and abuses of participation. In: Cornwall A ed. The Participation Reader. London, UK: Zed Books 2011:57-69.

29. Mathias K, Pillai P, Gaitonde R et al. Co-production of a pictorial recovery tool for people with psycho-social disability informed by a participatory action research approach—a qualitative study set in India. *Health Promot Int* 2020:35;486-99.

30. Robertson G. (2016). The global importance of including mental health carers in policy. *Institute for Development Studies, 117.*

31. De Weger E, Van Vooren N, Luijkx, KG et al. Achieving successful community engagement: a rapid realist review. *BMC Health Serv Res* 2018:18;1-18.

For peer review only

32. Rifkin S, Lewando-Hundt G, Draper A. Participatory approaches in health promotion and health planning: a literature review. *Health Development Agency,* 2000.
33. Klingberg, S. Adhikari, B., Draper, CE. Engaging communities in non-communicable disease research and interventions in low- and middle-income countries: a realist review protocol. *BMJ Open* 2021:11(7).
34. Wong G, Greenhalgh T, Westhorp G, et al. RAMESES publication standards: realist syntheses. *BMC Med* 2013:11;1-14.
35. Pawson R, Greenhalgh T, Harvey G et al. Realist review-a new method of systematic review designed for complex policy interventions. *J Health Serv Res Policy, 2005:10;*21-34.
36. Saul JE, Willis CD, Bitz J. et al. A time-responsive tool for informing policy making: rapid realist review. *Implement Sci* 2013:8;1-15.
37. Brown SL, Hussier M, Dalki SM. et al. Care planning: What works, for whom, and in what circumstances? A rapid realist review. *Qual Health Res* 2018:28;2250-66.
38. Goodman C, Dening T, Gordon AL. et al. Effective health care for older people living and dying in care homes: a realist review. *BMC Health Serv Res* 2016:*16*;1-14.
39. de Sousa Santos B. The End of Cognitive Empire: The Coming of Age of Epistemologies of the South. Duke University Press 2018.
40. World Health Organisation. Mental health and development: Targeting people with mental health conditions as a vulnerable group. 2020; https://www.who.int/publications-detail-redirect/9789241563949. (accessed 30/1/2021)
41. WHO. Mental health: new understanding, new hope. 2001; http://www.who.int/whr/2001/en. (accessed 1/2/2021)
42. JBI Institute. Critical appraisal tools, 2021; https://jbi.global/critical-appraisal-tools (accessed 1/3/2021)
43. Mathias K, Singh P, Butcher N. et al. Promoting social inclusion for young people affected by psycho-social disability in India – a realist evaluation of a pilot intervention. *Global Public Health* 2019; 14(12):1718-1732,

For peer review only

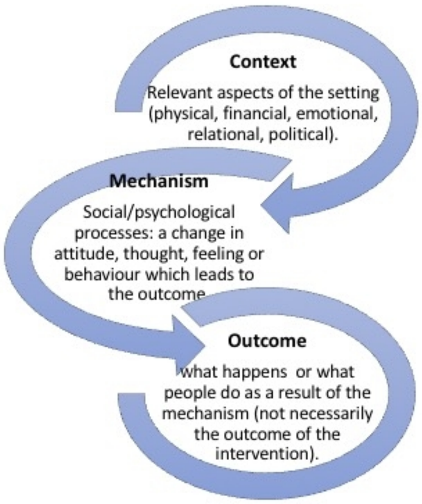

Figure 1_CMO configurations

338x190mm (54 x 54 DPI)

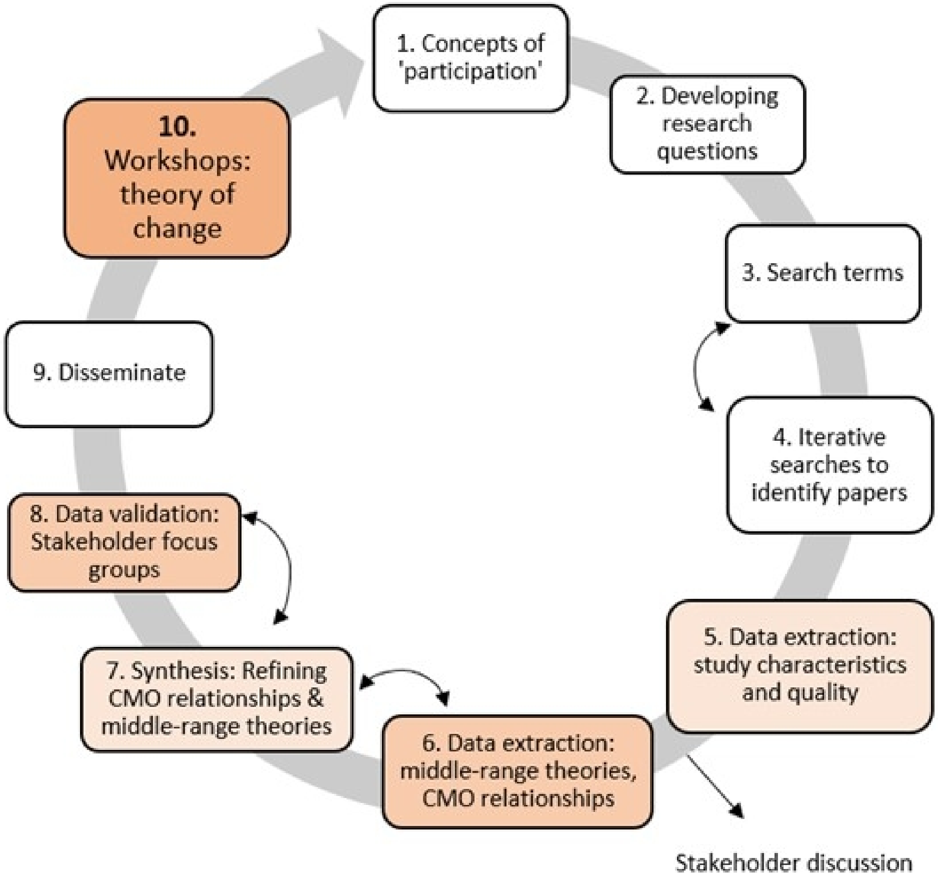

Figure 2_10 step review process

148x133mm (96 x 96 DPI)

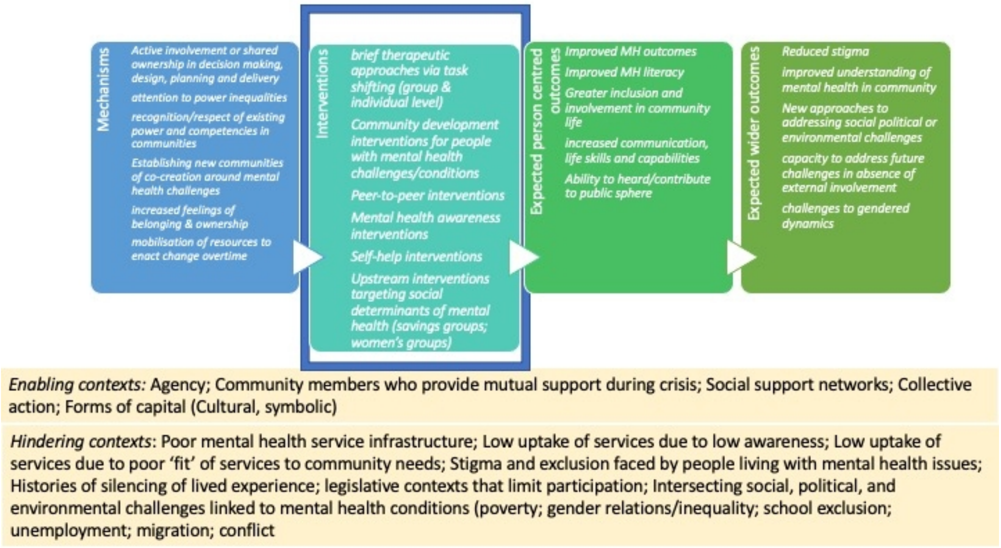

Figure 3_Programme theory

338x190mm (54 x 54 DPI)

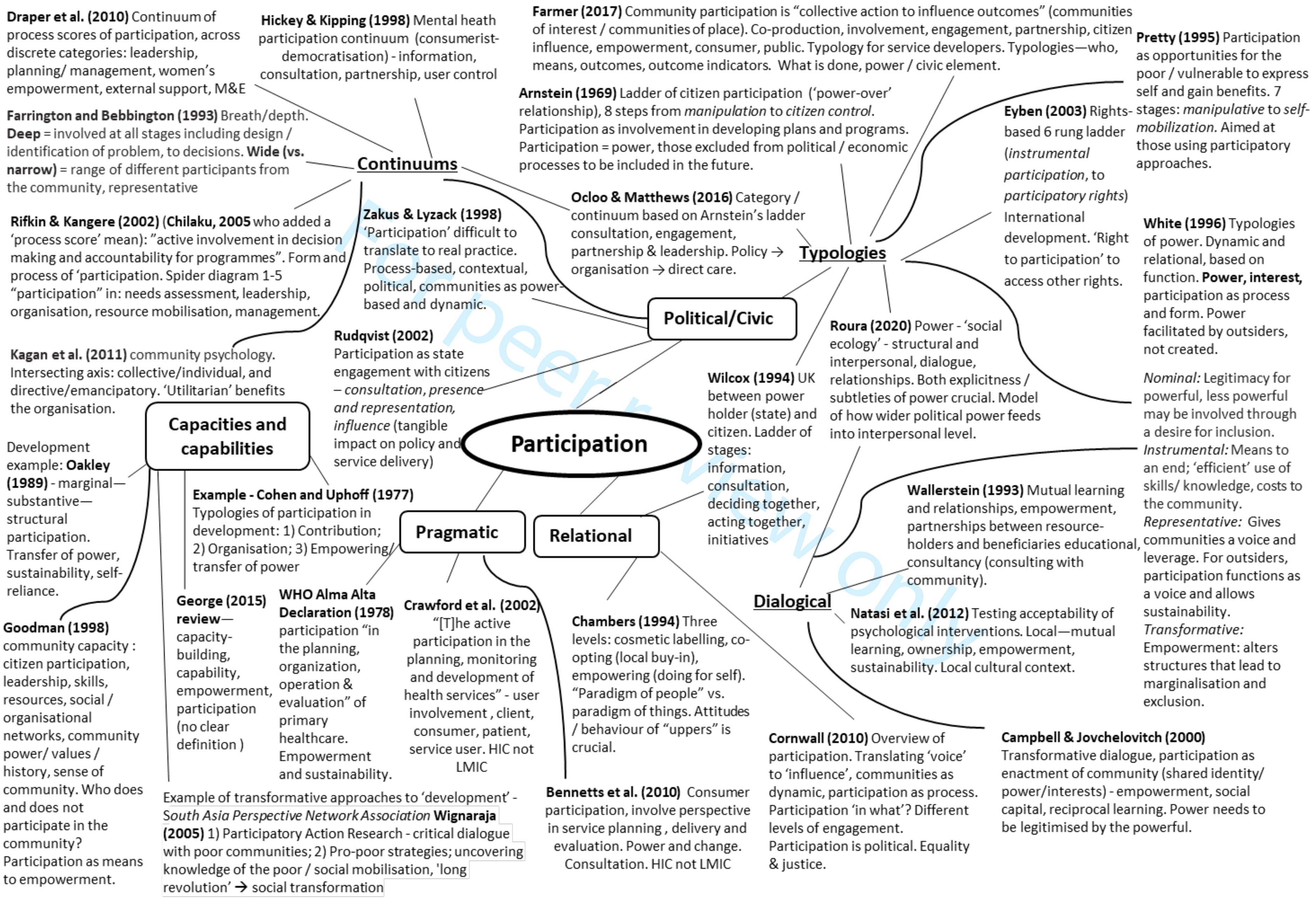
Participation
Continuums
Typologies
Political/Civic
Capacities and capabilities
Pragmatic
Relational
Dialogical
Draper et al. (2010) Continuum of process scores of participation, across discrete categories: leadership, planning/ management, women's empowerment, external support, M&E
Hickey & Kipping (1998) Mental heath participation continuum (consumerist-democratisation) - information, consultation, partnership, user control
Farrington and Bebbington (1993) Breath/depth. Deep = involved at all stages including design / identification of problem, to decisions. Wide (vs. narrow) = range of different participants from the community, representative
Rifkin & Kangere (2002) (Chilaku, 2005 who added a 'process score' mean): "active involvement in decision making and accountability for programmes". Form and process of 'participation. Spider diagram 1-5 "participation" in: needs assessment, leadership, organisation, resource mobilisation, management.
Zakus & Lyzack (1998) 'Participation' difficult to translate to real practice. Process-based, contextual, political, communities as power-based and dynamic.
Kagan et al. (2011) community psychology. Intersecting axis: collective/individual, and directive/emancipatory. 'Utilitarian' benefits the organisation.
Rudqvist (2002) Participation as state engagement with citizens – consultation, presence and representation, influence (tangible impact on policy and service delivery)
Farmer (2017) Community participation is "collective action to influence outcomes" (communities of interest / communities of place). Co-production, involvement, engagement, partnership, citizen influence, empowerment, consumer, public. Typology for service developers. Typologies—who, means, outcomes, outcome indicators. What is done, power / civic element.
Arnstein (1969) Ladder of citizen participation ('power-over' relationship), 8 steps from manipulation to citizen control. Participation as involvement in developing plans and programs. Participation = power, those excluded from political / economic processes to be included in the future.
Ocloo & Matthews (2016) Category / continuum based on Arnstein's ladder consultation, engagement, partnership & leadership. Policy → organisation → direct care.
Pretty (1995) Participation as opportunities for the poor / vulnerable to express self and gain benefits. 7 stages: manipulative to self-mobilization. Aimed at those using participatory approaches.
Eyben (2003) Rights-based 6 rung ladder (instrumental participation, to participatory rights) International development. 'Right to participation' to access other rights.
White (1996) Typologies of power. Dynamic and relational, based on function. Power, interest, participation as process and form. Power facilitated by outsiders, not created.
Nominal: Legitimacy for powerful, less powerful may be involved through a desire for inclusion.
Instrumental: Means to an end; 'efficient' use of skills/ knowledge, costs to the community.
Representative: Gives communities a voice and leverage. For outsiders, participation functions as a voice and allows sustainability.
Transformative: Empowerment: alters structures that lead to marginalisation and exclusion.
Roura (2020) Power - 'social ecology' - structural and interpersonal, dialogue, relationships. Both explicitness / subtleties of power crucial. Model of how wider political power feeds into interpersonal level.
Wilcox (1994) UK between power holder (state) and citizen. Ladder of stages: information, consultation, deciding together, acting together, initiatives
Development example: Oakley (1989) - marginal—substantive—structural participation. Transfer of power, sustainability, self-reliance.
Example - Cohen and Uphoff (1977) Typologies of participation in development: 1) Contribution; 2) Organisation; 3) Empowering/ transfer of power
Wallerstein (1993) Mutual learning and relationships, empowerment, partnerships between resource-holders and beneficiaries educational, consultancy (consulting with community).
Natasi et al. (2012) Testing acceptability of psychological interventions. Local—mutual learning, ownership, empowerment, sustainability. Local cultural context.
Goodman (1998) community capacity : citizen participation, leadership, skills, resources, social / organisational networks, community power/ values / history, sense of community. Who does and does not participate in the community? Participation as means to empowerment.
George (2015) review—capacity-building, capability, empowerment, participation (no clear definition)
WHO Alma Alta Declaration (1978) participation "in the planning, organization, operation & evaluation" of primary healthcare. Empowerment and sustainability.
Crawford et al. (2002) "[T]he active participation in the planning, monitoring and development of health services" - user involvement , client, consumer, patient, service user. HIC not LMIC
Chambers (1994) Three levels: cosmetic labelling, co-opting (local buy-in), empowering (doing for self). "Paradigm of people" vs. paradigm of things. Attitudes / behaviour of "uppers" is crucial.
Cornwall (2010) Overview of participation. Translating 'voice' to 'influence', communities as dynamic, participation as process. Participation 'in what'? Different levels of engagement. Participation is political. Equality & justice.
Campbell & Jovchelovitch (2000) Transformative dialogue, participation as enactment of community (shared identity/ power/interests) - empowerment, social capital, reciprocal learning. Power needs to be legitimised by the powerful.
Example of transformative approaches to 'development' - South Asia Perspective Network Association Wignaraja (2005) 1) Participatory Action Research - critical dialogue with poor communities; 2) Pro-poor strategies; uncovering knowledge of the poor / social mobilisation, 'long revolution' → social transformation
Bennetts et al. (2010) Consumer participation, involve perspective in service planning , delivery and evaluation. Power and change. Consultation. HIC not LMIC

Reporting checklist for protocol of a systematic review and meta analysis.

Based on the PRISMA-P guidelines.

Instructions to authors

Complete this checklist by entering the page numbers from your manuscript where readers will find each of the items listed below.

Your article may not currently address all the items on the checklist. Please modify your text to include the missing information. If you are certain that an item does not apply, please write "n/a" and provide a short explanation.

Upload your completed checklist as an extra file when you submit to a journal.

In your methods section, say that you used the PRISMA-Preporting guidelines, and cite them as:

Moher D, Shamseer L, Clarke M, Ghersi D, Liberati A, Petticrew M, Shekelle P, Stewart LA. Preferred Reporting Items for Systematic Review and Meta-Analysis Protocols (PRISMA-P) 2015 statement. Syst Rev. 2015;4(1):1.

		Reporting Item	Page Number
**Title**			
Identification	#1a	Identify the report as a protocol of a systematic review	1
Update	#1b	If the protocol is for an update of a previous systematic review, identify as such	N/A
**Registration**			
	#2	If registered, provide the name of the registry (such as PROSPERO) and registration number	16
**Authors**			
Contact	#3a	Provide name, institutional affiliation, e-mail address of all protocol authors; provide physical mailing address of corresponding author	1
Contribution	#3b	Describe contributions of protocol authors and identify the guarantor of the review	19

For peer review only

Amendments

	#4	If the protocol represents an amendment of a previously completed or published protocol, identify as such and list changes; otherwise, state plan for documenting important protocol amendments	N/A

Support

Sources	#5a	Indicate sources of financial or other support for the review	20
Sponsor	#5b	Provide name for the review funder and / or sponsor	20
Role of sponsor or funder	#5c	Describe roles of funder(s), sponsor(s), and / or institution(s), if any, in developing the protocol	20

Introduction

Rationale	#6	Describe the rationale for the review in the context of what is already known	3-8
Objectives	#7	Provide an explicit statement of the question(s) the review will address with reference to participants, interventions, comparators, and outcomes (PICO)	8

Methods

Eligibility criteria	#8	Specify the study characteristics (such as PICO, study design, setting, time frame) and report characteristics (such as years considered, language, publication status) to be used as criteria for eligibility for the review	11
Information sources	#9	Describe all intended information sources (such as electronic databases, contact with study authors, trial registers or other grey literature sources) with planned dates of coverage	10
Search strategy	#10	Present draft of search strategy to be used for at least one electronic database, including planned limits, such that it could be repeated	10-12
Study records - data management	#11a	Describe the mechanism(s) that will be used to manage records and data throughout the review	11
Study records - selection process	#11b	State the process that will be used for selecting studies (such as two independent reviewers) through each phase of the review (that is, screening, eligibility and inclusion in meta-analysis)	11-12
Study records - data	#11c	Describe planned method of extracting data from reports (such as	11-12

For peer review only

collection process		piloting forms, done independently, in duplicate), any processes for obtaining and confirming data from investigators	
Data items	#12	List and define all variables for which data will be sought (such as PICO items, funding sources), any pre-planned data assumptions and simplifications	11-12
Outcomes and prioritization	#13	List and define all outcomes for which data will be sought, including prioritization of main and additional outcomes, with rationale	N/A
Risk of bias in individual studies	#14	Describe anticipated methods for assessing risk of bias of individual studies, including whether this will be done at the outcome or study level, or both; state how this information will be used in data synthesis	13
Data synthesis	#15a	Describe criteria under which study data will be quantitatively synthesised	N/A
Data synthesis	#15b	If data are appropriate for quantitative synthesis, describe planned summary measures, methods of handling data and methods of combining data from studies, including any planned exploration of consistency (such as I2, Kendall's τ)	N/A
Data synthesis	#15c	Describe any proposed additional analyses (such as sensitivity or subgroup analyses, meta-regression)	N/A
Data synthesis	#15d	If quantitative synthesis is not appropriate, describe the type of summary planned	15-16
Meta-bias(es)	#16	Specify any planned assessment of meta-bias(es) (such as publication bias across studies, selective reporting within studies)	N/A
Confidence in cumulative evidence	#17	Describe how the strength of the body of evidence will be assessed (such as GRADE)	N/A

The PRISMA-P elaboration and explanation paper is distributed under the terms of the Creative Commons Attribution License CC-BY. This checklist was completed on 18. September 2021 using https://www.goodreports.org/, a tool made by the EQUATOR Network in collaboration with Penelope.ai

For peer review only

BMJ Open

Participatory mental health interventions in low and middle income countries: a realist review protocol

Journal:	*BMJ Open*
Manuscript ID	bmjopen-2021-057530.R1
Article Type:	Protocol
Date Submitted by the Author:	10-Feb-2022
Complete List of Authors:	Heap, Cheyann ; University of York, Department of Social Work and Social Policy<br>Jennings, Hannah; University of York; University College London<br>Mathias, Kaaren; University of Canterbury, School of Health Sciences; Burans, Herbertpur Christian hospital<br>Gaire, Himal; Centre for Mental Health Counselling (CMC)<br>Gumbonzvanda, Farirai; Rozaria Memorial Trust<br>Gumbonzvanda, Nyaradzayi; Rozaria Memorial Trust<br>Gupta, Garima; Burans, Herbertpur Christian hospital<br>Jain, Sumeet; The University of Edinburgh School of Social and Political Science<br>Maharjan, Bidya; Chhahari Nepal for Mental Health (CNMH)<br>Maharjan, Rakchhya; Chhahari Nepal for Mental Health (CNMH)<br>Maharjan, Sujen Man; Chhahari Nepal for Mental Health (CNMH)<br>Mahat, Pashupati; Centre for Mental Health and Counselling<br>Pillai, Pooja; Burans, Herbertpur Christian hospital<br>Webber, Martin; University of York, Department of Social Policy and Social Work<br>Wright, Jerome; University of York<br>Burgess, Rochelle; UCL Institute for Global Health
**Primary Subject Heading**:	Mental health
Secondary Subject Heading:	Global health
Keywords:	MENTAL HEALTH, PUBLIC HEALTH, QUALITATIVE RESEARCH

SCHOLARONE™ Manuscripts

Participatory mental health interventions in low and middle income countries: a realist review protocol

Authors:

Cheyann J Heap[1]; Hannah Maria Jennings[2 3 4 *]; Kaaren Mathias[5 6]; Himal Gaire[7]; Farirai Gumbonzvanda[8]; Nyaradzayi Gumbonzvanda[8]; Garima Gupta[5]; Sumeet Jain[9] ; Bidya Maharjan[10]; Rakchhya Maharjan[10]; Sujen Man Maharjan[10]; Pashupati Mahat[7]; Pooja Pillai[5]; Martin Webber[1]; Jerome Wright[2]; Rochelle A Burgess[4]

[1] Department of Social Work and Social Policy, University of York, UK

[2] Department of Health Sciences, University of York, UK

[3] Hull York Medical School, University of York, UK

[4] Institute for Global Health, University College London, UK

[5] Burans, Herbertpur Christian hospital, India

[6] School of Health sciences, University of Canterbury, New Zealand

[7] Centre for Mental Health and Counselling (CMC), Nepal

[8] Rozaria Memorial Trust (RMT), Zimbabwe

[9] School of Social and Political Science, University of Edinburgh, UK

[10] Chhahari Nepal for Mental Health (CNMH), Nepal

*corresponding author: hannah.jennings@york.ac.uk Department of Health Sciences, University of York, Heslington, York YO10 5DD

Word count: 4,004 words

For peer review only

ABSTRACT

Introduction: The launch of the Movement for Global Mental Health brought longstanding calls for improved mental health interventions in low-and middle-income countries (LMICs) to centre stage. Within the movement, the participation of communities and people with lived experience of mental health problems is argued as essential to successful interventions. However, there remains a lack of conceptual clarity around 'participation' in mental health interventions with the specific elements of participation rarely articulated. Our review responds to this gap by exploring how 'participation' is applied, what it means and what key mechanisms contribute to change in participatory interventions for mental health in LMICs.

Methods and analysis: A realist review methodology will be used to identify the different contexts which trigger mechanisms of change, and the resulting outcomes related to the development and implementation of participatory mental health interventions, that is: what makes participation *work* in mental health interventions in LMICs, and *why*? We augment our search with primary data collection in communities who are the targets of global mental health initiatives, to inform the production of a programme theory on participation for mental health in LMICs.

Ethics and dissemination: Ethical approval for Focus Group Discussions (FGDs) was obtained in each country involved**.** FGDs will be conducted in line with WHO safety guidance during the covid-19 crisis. The full review will be published in an academic journal, with further papers providing an in-depth analysis on community perspectives on participation in mental health. The project findings will also be shared on a website, in webinars and an online workshop.

Keywords: participation, mental health interventions, realist review, LMICs, community, empowerment, involvement

For peer review only

STRENGTHS AND LIMITATIONS

- The review is strengthened by stakeholder involvement - FGDs with people with mental health problems and carers in LMICs.
- The review is theory-driven, using concepts of participation from academic literature and applied practice as a framework.
- The review is limited to published academic papers in English; there may be further insights available in grey literature and in other languages.

For peer review only

INTRODUCTION

Globally, mental ill health is a leading cause of disability[1]. Socio-structural factors are increasingly recognised as causes and consequences of poor mental health[2-3]. In 2007, *The Lancet's* pivotal 'Global Mental Health' series highlighted global inequalities in mental health provision, particularly in low-and-middle-income countries (LMICs)[4], marking the beginning of what is now commonly referred to as the *Movement for Global Mental Health*[5]. The movement advocates evidence-based interventions, informed by a human rights approach, with the aim to scale up mental health services. However, these efforts operate primarily within an essentially biomedical framework that eschews attention to structural drivers of distress in favour of pragmatics in clinical care. The movement has faced extensive critique because of this approach, with a special section of Transcultural Psychiatry in 2012 arguing for a greater emphasis of engagement with local communities within mental health to overcome these limitations[6]. For example, when communities actively participate in the governance, design or delivery of mental health interventions they can be more acceptable and relevant to local needs as well as more cost-effect and higher quality[7]. Furthermore, depending on the social processes involved, participation can be empowering and even transformative to communities[8-9]. However, an inattention to power dynamics when working on 'participation' run the risk of exacerbating existing patterns of exclusion and reinforcing inequities[8].

As a group of researchers and practitioners, we are interested in the theory and application of participation in global mental health interventions in LMICs for two key reasons: First, we argue that how people in LMICs define participation in mental health interventions or more broadly, matters to programmes. Second, we argue that participation in mental health interventions creates platforms for transformation in the lives of people it seeks to benefits *if* attention is paid to key processes.

For peer review only

Theories of participation

Debates around the active involvement of communities in localities in LMICs – referred to as ‘participation’, ‘community engagement’ and ‘mobilisation’ – are important and related principles can be found within the broader fields of global health and international development. The diversity of approaches relating to the term, also lead to its evolution into what some argue as a benign concept, a term with wide reaching boundaries which enable its wide ranging applications[10]. There are different perspectives on how ‘participation’ is achieved and for what purposes participation is implemented[9]. For example, Campbell and Jovchelovitch suggest that participation is a mechanism for negotiating social identity, and participatory power thus emerges as “a space of possible action”[11]. Others suggest that mental health ‘consumer participation’ must include the power to take meaningful action through orchestrating changes in service planning, delivery and intervention[12-13]. What action looks like is dependant on the specific theoretical approach applied in a certain context.

Participatory theories can be broadly separated into *typologies* (categories, often set as ‘stages’) or *continuums* (spectrums). ‘Ladder’ or stage typologies are common in civic, political understandings of power. Arnstein’s famous (1969) ladder of citizen participation has eight rungs from *manipulation* to *citizen control*[14]. Similarly, Goetz and Gaventa range from *consultation* (information sharing) to *influence* (tangible impact on policy making and service delivery)[15], and Wilcox considers collaboration between citizen and state from *information* to *initiatives*[16]. Pretty frames ‘participation’ as opportunities for the poor to gain benefits, from *manipulative* to *self-mobilization*[17], whereas Eyben’s rights-based, six-rung ladder (*instrumental participation* to *participatory rights*) suggests that the right to participation facilitates access to other human rights[18]. These ladders/stages have several commonalities: on lower rungs people have little power, and any inclusion of their voices is tokenistic and does not lead to tangible change. The top rungs are interventions designed and led by the community. However, ladders do not effectively accommodate the dynamic, contextual and relational

For peer review only

aspects of power in participation. Additionally, a fully citizen-led approach may not always be possible or even desirable.

Continuums can accommodate shifts in participation and power status. In community development, Draper et al. created a process-based continuum of participation across five categories including external support, leadership and women's empowerment[19]. Bebbington and Farrington's continuum focuses on participation breadth (representativeness of the full community), and depth (extent of meaningful involvement)[20]. Similar to participation ladders, Hickey and Kipping's mental health participation continuum ranges from *information* to *user control*, with a shift from passive 'consumerism' to 'democratisation'[21]. Likewise, Rifkin and Kangere argue participation ranges from people as passive receivers to active decision makers, acknowledging change over time [22].

Some approaches emphasise the dialogical nature of participation. Campbell and Jovchelovitch take a Freirean approach, noting how transformative dialogue can lead to reciprocal growth and learning between 'the powerful' and 'the community'[23]. Wallerstein also highlights the capacity for mutual learning and reciprocal relationships, with partnerships between resource-holders and beneficiaries leading to transformational change for both[24]. The South Asia Perspective Network Association goes further to emphasise deliberately 'pro-poor' strategies, where through a dialogic approach, sustained participatory action and new social movements lead to wider social transformation[25]. Nastasi et al.'s Participatory Intervention Model applies this idea to mental health interventions, noting the importance of relationships, changing interpersonal and power dynamics, and matching knowledge and support to the needs of the community[26]. Indeed, communities are constantly shifting and have their own internal power dynamics[27]. White takes account of this in a relationally-based typological approach, which accounts for shifting priorities and dynamics between the community and those with power[28]. This typology ranges from *nominal* (tokenistic involvement), to *transformative* (empowerment).

For peer review only

Supplementary file 1 shows our mind map bringing together key debates in participation based around who it is for, how it is used, and the impact (if any) on participatory approaches. The mind map is intended to be comprehensive but not exhaustive. It was supplemented by discussion on the lived experiences of practitioners in the team and the participatory work of their organisations[29-30]. We conceptualise 'participation' in mental health as *the active involvement of people affected by interventions or targeted action (including wider research projects).* This can include a range of actors: service users, carers, providers, and wider community members. Ultimately, they all contribute to the intervention in some capacity, and no group are merely passive recipients of an outcome. This includes involvement and ownership over various phases of concept, design, implementation, and evaluation stages of interventions. Notably, our approach recognises a range of dynamics and interest which reflect on how the following factors shape participation including: interests and motivations, socio-economic context, influence, and structural and interpersonal power.

Realist Review on Participation on Mental Health in LMIC

To date there have been reviews of community engagement and participatory health approaches in LMICs[31-32] including an ongoing realist review on community engagement in non-communicable disease interventions and research[33], however to the authors' knowledge there has been no specific review which interrogates participatory approaches to mental health across LMICs. The current review seeks to address this gap exploring the following questions: In LMIC settings, what is the nature of participatory approaches for mental health improvement? Who are the targets of participation in mental health research and practice? What factors contribute to their success or failure?

Our investigation applies a realist methodology, which has a similar interest in "what works for these particular people in particular contexts"[31,34]. Realists reviews combine theory and empirical observation and seek to understand how and why change is facilitated[31,33,34, 35]. This review will be

For peer review only

used to identify mechanisms of change and their related contexts, which will support the development of theory to further our understandings of how participation is used in the design and implementation of mental health interventions.

Aim and research questions

The aim of the review is to interrogate existing literature relating to participatory mental health interventions in low- to middle-income countries, to consider how, for whom, and under what conditions participatory approaches work. Across two stages of data extraction, we will seek to answer the following questions:

1. Why and for whom has participation been used in mental health interventions in LMIC?
2. How and to what extent has participation been operationalised in research versus implementation?
3. What are the mechanisms of action of participation and how are they linked to local contexts?
4. Why, how and under what circumstances does community participation in mental health interventions lead to improved mental health?

METHOD

This review was designed adapting Saul et al's suggested 10 steps for a realist review[36]. Realist reviews often involve developing an initial programme theory, which is later refined through the development of middle-range theories of what works[36-38]. Middle-range theories are general enough to be transferred to other projects, but concrete and specific enough to apply to practice[34]. In generating or exploring theory, Context, Mechanism and Outcomes are crucial **[Figure 1: CMO configurations]**.

For peer review only

When relations between these factors are explored, they create 'CMO configurations' and are developed in response to particular research questions[38].

Figure 1: CMO configurations

As not all papers will have enough detail to infer theories and extract CMO configurations, data extraction will be in two stages. Stage one will extract surface-level study characteristics, and quality. Stage two will complete a deeper analysis of the highest quality papers, from which CMO configurations will be extracted to build our middle-range theories. To counter the western hegemonic knowledge structures that shape much of the peer reviewed literature[39] we have added specific stakeholder consultations in our review, to combine and include embodied knowledges held by people with mental health difficulties, carers and community members. Consulting stakeholders as part of the review is consistent with the realist methodology.[31,33] We assess their views of participation and involvement as it relates to mental health, at two stages in the review (see step 7). The 10 steps as we have applied them in our study are described in more detail below **[Figure 2: 10 step review process].**

Figure 2: 10 step review process

We will report the results of the review according to the Realist and Meta-narrative Evidence Syntheses: Evolving Standards (RAMESES) quality and publication standards.[34]

Concepts of 'participation' and developing research questions (steps 1 and 2)

A mind map of different concepts of 'participation' **[Supplementary file 1]** was created between January and March 2021. This involved reading research literature on participation, particularly on LMICs and from the academic fields of development, health and human rights. This was supplemented by monthly meetings between the entire research group, to discuss emerging themes and link the research to the applied practice of team members. Team members, across LMICs and high-income countries, represent varied ethnic backgrounds and disciplinary backgrounds in mental health,

For peer review only

medicine, social work, mental health nursing, psychology, academia, and non-governmental organisations. Key debates from the mindmap and group discussion were used to develop the four research questions.

Based on this initial conceptual review and discussions, project PI (RB) drafted an initial programme theory (IPT) and model which was discussed and refined within the research group **[Figure 3: programme theory]**. The IPT guided the remainder of the review process and will be refined in light of the findings of this review (see step 9).

Our IPT considers contexts at multiple levels, and is purposefully broad. The IPT suggests, that overall, individuals who live with or are at risk of developing mental health conditions in the global south (LMIC) [who] do so because of a number of daily realities across various settings with relations to mental health needs (contexts barriers). We postulate that these wider limiting contexts will include: poor mental health service infrastructure; low uptake of services due to low awareness of services and due to a poor 'fit' of services to the needs of communities; stigma and exclusion faced by people living with mental health issues; histories of silencing of lived experience within the mental health space; and intersecting social, political, and environmental challenges that link to mental health conditions. The second level of contexts we are interested in, are those which may contribute positively to the impact of interventions and mental health (enabling contexts). These include various forms of capital, agency, collective mobilisation and supportive community partnerships.

We believe, that if participatory approaches to mental health meet the parameters of *meaningful participation* (which we defined as transformative participation outlined by White's framework[28]), then this would activate a series of mechanisms that would lead to positive outcomes on mental health conditions, as well as a wider impacts on societal factors linked to poor mental health (addressing hindering contexts). We believe this would be the case across various population groups

For peer review only

at risk for developing mental health conditions (who). This is because transformative participation creates opportunities for participants to change the environments that place good mental health at risk, as well as increase access to better services through various pathways such as the production of new communities of practice and support networks.

We imagine the potential outcomes could occur at two levels. At the individual level, this may include: improved mental health outcomes; improved access to services, improved mental health literacy, a reduction in experiences of exclusion. At wider societal levels, it could include: a reduction in harmful social, political and environmental drivers of distress (i.e. reduced poverty; reduced gender discrimination; improved living conditions) and increased voice and ownership for people with lived experience in wider society. We believe that when meaningful participation [transformative] is not present, positive impacts may occur, but only for individual outcomes. Ultimately, this theory helps us to determine not only the mechanisms through which participatory interventions may work, but the ability for participatory interventions to be activated in the presence of enabling contexts. Also, our IPT allows us to account for the fact that transformative participatory interventions themselves may work to change the contexts that trigger or block the mechanisms which drive better mental health. As such, we don't imagine a hard line between interventions and mechanisms, but will allow our literature review and stakeholder engagement to help illuminate these dynamics in the contexts of interventions that have transformative interests at their core.

Figure 3: Programme theory

Search terms and identifying papers (steps 3 and 4)

Search terms and databases searched were discussed in team meetings in early 2021. Searches are being carried out in the databases: Scopus, Medline/PsychINFO, ASSIA, CINAHL, EMBASE and JSTOR. If additional studies are identified in the references or through the team they will be considered for

For peer review only

screening. **Table 1** shows our search terms. **Supplementary file 2** gives details for the full search strategy.

Table 1: Literature search terms

The overall search formula included three umbrella concepts: *(('Participation') AND ('Mental health') AND ('LMICs')).* The terms under each umbrella (e.g. 'Participation') were separated by the Boolean operator *OR***		
'Participation'	**'Mental Health'****	**'LMICs' (Low to Middle Income Countries)**
• participat*<br>• empower*<br>• community<br>• community?led<br>• co-designed<br>• inclusion<br>• inclusive<br>• capacity building<br>• capabilities<br>• engagement<br>• consultation<br>• co-produc*<br>• peer-led<br>• peer?to?peer<br>• task?shifting<br>• task?switching	• psychological disorder<br>• psychological problem<br>• Psychological illness<br>• Psychological distress<br>• psychiatric disorder<br>• psychiatric problem<br>• psychiatric illness<br>• psychiatric distress<br>• psychosocial disability<br>• mental illness<br>• mental health<br>• SMI [Serious Mental Illness]	• Developing countr*<br>• low?income countr*<br>• middle?income countr*
**phrases of more than one word were put into "double quotes" for the search<br>***There are a wide range of conceptions of mental health, including medical ("mental illness") and psychosocial[40]. We tried to accommodate this variety in our search terms.		

Screening and study selection

Instead of limiting to a particular definition of 'participation', this review will take a pragmatic approach. It will include all papers which either: a) claim to be participatory, or b) clearly include participation (the active involvement of people with mental health problems and their supporters). We will include studies located in low- and middle- income countries (as according the World Bank definition) that are related to a mental health intervention. We use a broad definition of mental health

For peer review only

as defined by the WHO and draw on their list of mental and neurological disorders[40]. We define interventions as programmes or policies that are implemented with the aim to change outcome/s. **Table two** shows our inclusion and exclusion criteria.

Table 2: Inclusion and exclusion criteria

Inclusion Criteria	Exclusion criteria
Located in low- and middle- income countries;<br><br>Health and development projects/ interventions addressing mental health;<br><br>Intervention, or research study about an intervention, which claims to be participatory OR clearly demonstrates the active involvement of the target population in concept, design, implementation, or evaluation;<br><br>Any 'level' of intervention including individual, group, and systemic interventions such as national and international projects;<br><br>Any method – qualitative, quantitative, case study;<br><br>Participants are people with mental health problems, their unpaid carers, or other people from the community such as local health workers or laypeople;<br><br>On or after 2001, based on the World Health Report which presented community-based mental health services as critical[41]	'Participation' does not include the active involvement of people from the target community (e.g. laypeople, local professionals, people with mental health problems, carers);<br><br>Not about an intervention ('intervention' = support group, a specific mental health policy, training)

Possible relevant papers identified during the searches will be uploaded onto the software Ryaan for title and abstract screening. Title and abstract screening will be conducted blinded by members of the

For peer review only

research team, to ensure consistency 10% of studies screened by two members of the research team. Full text screening will be completed by the whole research team with members working in pairs and the papers divided among the team. Any uncertainties throughout the process will be discussed within the team at regular team meetings.

Data extraction and quality appraisal (steps 5 and 6)

There will be two stages of data extraction. The first stage will answer the first two research questions of the review.:

1. Why and for whom has participation been used in mental health interventions in LMIC?
2. How and to what extent has participation been operationalised in research versus implementation?

At the end of the first stage, the highest-quality papers with the most detail about theories of participation and context, mechanisms and outcomes will be identified and taken forward to the second stage. This more in-depth analysis and CMO configuration extraction will answer the final two questions:

3. What are the mechanisms of action of participation and how are they linked to local contexts?
4. Why, how and under what circumstances does community participation in mental health interventions lead to improved mental health?

Stage one

As with the full text screening data extraction will be completed in pairs by the research team. A data extraction tool will be developed, and refined as relevant data are extracted. Extracted data will likely include: study aims, methods, why participation is used; concept and theory of 'participation'; target

For peer review only

population and stakeholders; how participation is 'done'; outcomes of intervention; level of participation; analysis of power. For quality, a realist review asks whether papers are *relevant* (to the research question) and *rigorous* (of good-enough quality to make a meaningful contribution)[34]. The data extraction tool will include whether papers are 'low, medium and high' relevance. For 'rigour', as we are including all methods (case studies, qualitative and quantitative), specific quality checklists will be selected based on the nature of the papers, primarily drawing from the Joanna Briggs Institute Critical Appraisal tools[42]. Uncertainties as to the data extraction, relevance and rigour will be discussed in the team meetings and Research Associate (CH) and principle investigators (RB and HJ) will do final quality checks.

Stage two

Papers of 'high quality' – rigorous, relevant, and with sufficient information to extract CMO configurations - will be taken forward for the second stage of review. Potential studies for this stage of data extraction will be identified by the research team during stage one. All studies identified and those marked as 'high' for relevance will be considered. Three members of the research team (CH, RB and HJ) will read the shortlisted paper and agree through consensus whether they should go through to the next round of the review. They will record and report back to the wider team the reasoning for the decisions. The criteria for studies to go through to the next round of review include: information and discussion about participation, enough detail to extract information on CMO configurations.

Focus Group Discussions (step 7)

There will be two rounds of stakeholder consultations through focus group discussions, across four sites in three countries (Nepal, India and Zimbabwe), organised by members of the network who work in participation and mental health (BM, RM, SMM, PM, PP, NG, FG, GG). The groups will include people affected by mental health difficulties, carers and community members.

For peer review only

There will be two rounds of FGDs. The first round of FGDs will explore understandings of participation and contributed to the IPT. In the second round of FGDs a summary of data so far from the literature review will be presented in order to get feedback and reflections from the participants, and will further contribute to the refinement of the programme theory. Findings of the FGDs will be compared and integrated with findings from the data extraction, to help theorise 'what works and how', in the way that is considered most participatory in the context. Findings from the FGDs will be reported in our final write-up, dissemination and revision of our IPT (steps 9 and 10).

Data synthesis and CMO configurations (step 8 and 9)

Stage one

The first stage of data synthesis based on the first round of data collection will be narrative, based around the first three research questions. It will report on the breadth and scope of participation as utilised in mental health settings in LMICs. Comparisons will be made with the FGDs and their understanding of participation.

Stage two

The second round of data synthesis is theory-focused and iterative process based on the 'high quality papers'. Data synthesis will take the following process:

- Taking our 'high quality' papers we will extract CMO configurations related to participation from each paper. This will be an iterative process where researchers will work in groups to discuss and extract the configurations. They will consider the evidence for the relationships between Context, Mechanisms and Outcomes relating to participation before finalising the CMO configurations.

For peer review only

- CMO configurations will be combined and refined to identify 'enabling contexts', the types of 'enabling mechanisms' they trigger and their possible outcomes (mid-range theories). Again this will be an iterative process involving the whole network.
- Based on these findings and the FGD analysis we will revise our IPT.

We plan to undertake this process as a team, involving workshops led by KM (who has experience in realist methodology)[43]. One workshop will develop our initial CMO configurations based on the 'high quality papers'. Subsequent team meetings will allow refining of CMO configurations, comparing them against a thematic analysis of FGDs. As theories and CMO configurations are gradually refined, the process will be carefully documented to justify changes and ensure a rigorous and transparent process. Overall, the synthesis should be such that the weaknesses (or omissions) in one particular paper from the review are accommodated for by the strengths of other papers. Once the review has been completed, we will have a set of middle-range theories, informed by the embodied knowledge of everyday citizens who are often the targets of mental health interventions in LMICS. The theories explain how participatory mental health interventions create changes in local context (C), to trigger socio-psychological mechanisms (M) that lead to new behaviour outcomes (O) in communities of interest. Final CMO configurations will be used to refine the initial programme theory by RB and HJ which will be feedback and refined by the wider group.

Dissemination and workshop (step 10)

It is anticipated that the current realist review will create a strong theoretical and practice-based foundation for future work in participatory research and practice for mental health in LMIC settings, and other environments of adversity. It will also directly shape the efforts of our network for participatory mental health in LMICs. We will disseminate our findings within a workshop series, this will allow us to expand our network by welcoming a range of interested stakeholders including people with mental health problems, paid and unpaid carers, practitioners, academics and policymakers. We

For peer review only

also plan to disseminate our findings though peer review papers, and blogs written in accessible and local languages in each of our country settings.

Patient and public involvement

As part of the review we will have planned stakeholder consultations with people with mental health difficulties, carers and community members in three LMICs (Nepal, India, Zimbabwe). The consultations are an important part of the review and will contribute to our refined programme theory. As part of our dissemination we will organise workshops with people with mental health difficulties, carers, practitioners and community members in LMICs.

ETHICS AND DISSIMINATION

Ethical approval for the study received from the University of York Health Science and Research Governance committee (HSRGC/2021/438/F) and we have received ethical approval from the relevant bodies of the countries involved (Nepal Health Research Council, 3026; Emmanuel Hospital Association Institutional Ethics Committee, 254; UCL Ethics Board 127/002 and The Women's University in Africa 02/2020). The review has been registered with PROSPERO (ID number CRD42021241787). The full review is intended to be published in an academic journal, with further papers providing an in-depth analysis on community perspectives on participation. The project findings will also be shared on a website, in webinars and an online workshop.

AUTHORS CONTRIBUTIONS

RB and HJ initiated the review. All members of the network contributed toward the mind-map and conception of the protocol. Following discussions with the whole team CH designed and drafted the initial protocol, which was subsequently reviewed by BM, KM, PM, MW, RM, SM, JW, HJ and RB. BM, PM, KM, GG, PP, FG, NG and CH have taken a lead in planning the FGDs. HJ, RB, BM, PM, KM and PP

For peer review only

have organised ethical approval in respective sites. RB and HJ finalised the protocol. All authors approved the final manuscript (CH, HJ, HG, FG, NG, GG, SJ, BM, RM, SM, PM, KM, PP, WM, JW, RB).

COMPETING INTERESTS STATEMENT

There are no competing interests.

FUNDING STATEMENT

This work was supported by Global Challenges Research Fund Pump Priming Award funded through the UKRI-MRC (2019-2020/2020-21 H0027804)

REFERENCES

1. Patel VP, Saxena S, Lund C. et al. (2018).The *Lancet* Commission on mental health and sustainable development. *The Lancet Commissions* 2018; 392(10157): 1553-1598.
2. Johnson S. Social interventions in mental health: a call to action. *Soc Psychiatry Psychiatr Epidemiol* 2017;52: 245-247.
3. Burgess R, Jain S, Petersen I. et al. Social interventions: a new era for global mental health? *Lancet Psychiatry* 2020;7:118-9.
4. Horton R. Launching a new movement for mental health. *Lancet* 2007;370:806.
5. Cooper S. Global mental health and its critics: moving beyond the impasse. *Crit Public Health* 2016;4:355-8.
6. Campbell C, Burgess R. The role of communities in advancing the goals of the Movement for Global Mental Health. *Transcult Psychiatry* 2012;49:379-95.
7. Ottmann G, Laragy C, Allen J, Feldman P. Coproduction in Practice: Participatory Action Research to Develop a Model of Community Aged Care. *Systemic Practice and Action Research* 2011;24(5):413-27.
8. George AS, Mehra V, Scott K. et al. Community participation in health systems research: a systematic review assessing the state of research, the nature of interventions involved and the features of engagement with communities. *PLoS One* 2015;10:p.e0141091.

For peer review only

9. Farmer J, Taylor J, Stewart E. et al. Citizen participation in health services co-production: a roadmap for navigating participation types and outcomes. *Aust J Prim Health* 2018;23:509-15.

10. Cornwall, A.. Unpacking 'Participation': models, meanings and practices. *Community Development Journal* 2008;*43*(3), 269-283.

11. Campbell C, Jovchelovitch S. Health, community and development: towards a social psychology of participation. *J Community Appl Soc Psychol* 2000;10:255-70

12. Bennetts W, Cross W, Bloomer M. Understanding consumer participation in mental health: Issues of power and change. *Int J Ment Health Nurs* 2011;20:155-164.

13. Crawford MJ, Rutter D, Manley C. et al. Systematic review of involving patients in the planning and development of health care. *BMJ* 2002*;*325:1263-8.

14. Arnstein SR. (1969). A ladder of citizen participation. *J Am Inst Plann,* 1969*;*35:216-224.

15. Goetz M, Gaventa J. Bringing citizen voice and client focus into service delivery. Brighton, England: *Institute of Development Studies* 2001.

16. Wilcox D. Community participation and empowerment: putting theory into practice. *RRA Notes* 1994;21:78-82.

17. Pretty J. Participatory learning for sustainable agriculture. World Development 1995;23:1247-63.

18. Eyben R. The rise of rights: rights-based approaches to international development. *IDS Policy Briefing* 2003:17;1-4.

19. Draper AK, Hewitt G, Rifkin S. Chasing the dragon: developing indicators for the assessment of community participation in health programmes. *Soc Sci Med* 2010:71;1102-9.

20. Bebbington A, Farrington J. Governments, NGOs and agricultural development: perspectives on changing inter-organisational relationships. *J Dev Stud* 1993*:*29;199-219.

21. Hickey G, Kipping C. Exploring the concept of user involvement in mental health through a participation continuum. *J Clin Nurs,* 1998:7;83-8.

22. Rifkin SB, Kangere M. What is participation? In: Hartley S (eds.) Community-based rehabilitation (CBR) as a participatory strategy in Africa. Ithaca, NY: Cornell University 2002:37-49.

23. Campbell C, Jovchelovitch S. Health, community and development: Towards a social psychology of participation. *J Community Appl Soc Psychol* 2000:10;255-70.

24. Wallerstein N. Empowerment and health: the theory and practice of community change. *Community Dev J* 1993:28;218-27.

25. Wignaraja P. Pro-poor Growth and Governance in South Asia—Decentralisation and Participatory Development. *The Pakistan Development Review* 2005:44;1159–1171)

26. Nastasi BK, Varjas K, Schensul SL. et al. The Participatory Intervention Model: A framework for conceptualizing and promoting intervention acceptability. *Sch Psychol Q* 2000:15;207-32.

For peer review only

27. Zakus D, Lyzack C. Revisiting community participation. *Health Policy Plan* 1998:13; 1-6.
28. White, S. Depoliticizing development: the uses and abuses of participation. In: Cornwall A ed. The Participation Reader. London, UK: Zed Books 2011:57-69.
29. Mathias K, Pillai P, Gaitonde R et al. Co-production of a pictorial recovery tool for people with psycho-social disability informed by a participatory action research approach—a qualitative study set in India. *Health Promot Int* 2020:35;486-99.
30. Robertson G. (2016). The global importance of including mental health carers in policy. *Institute for Development Studies, 117.*
31. De Weger E, Van Vooren N, Luijkx, KG et al. Achieving successful community engagement: a rapid realist review. *BMC Health Serv Res* 2018:18;1-18.
32. Rifkin S, Lewando-Hundt G, Draper A. Participatory approaches in health promotion and health planning: a literature review. *Health Development Agency,* 2000.
33. Klingberg, S. Adhikari, B., Draper, CE. Engaging communities in non-communicable disease research and interventions in low- and middle-income countries: a realist review protocol. *BMJ Open* 2021:11(7).
34. Wong G, Greenhalgh T, Westhorp G, et al. RAMESES publication standards: realist syntheses. *BMC Med* 2013:11;1-14.
35. Pawson R, Greenhalgh T, Harvey G et al. Realist review-a new method of systematic review designed for complex policy interventions. *J Health Serv Res Policy, 2005:10;*21-34.
36. Saul JE, Willis CD, Bitz J. et al. A time-responsive tool for informing policy making: rapid realist review. *Implement Sci* 2013:8;1-15.
37. Brown SL, Hussier M, Dalki SM. et al. Care planning: What works, for whom, and in what circumstances? A rapid realist review. *Qual Health Res* 2018:28;2250-66.
38. Goodman C, Dening T, Gordon AL. et al. Effective health care for older people living and dying in care homes: a realist review. *BMC Health Serv Res* 2016:*16*;1-14.
39. de Sousa Santos B. The End of Cognitive Empire: The Coming of Age of Epistemologies of the South. Duke University Press 2018.
40. World Health Organisation. Mental health and development: Targeting people with mental health conditions as a vulnerable group. 2020; https://www.who.int/publications-detail-redirect/9789241563949. (accessed 30/1/2021)
41. WHO. Mental health: new understanding, new hope. 2001; http://www.who.int/whr/2001/en. (accessed 1/2/2021)
42. JBI Institute. Critical appraisal tools, 2021; https://jbi.global/critical-appraisal-tools (accessed 1/3/2021)

For peer review only

43. Mathias K, Singh P, Butcher N. et al. Promoting social inclusion for young people affected by psycho-social disability in India – a realist evaluation of a pilot intervention. *Global Public Health* 2019; 14(12):1718-1732,

For peer review only

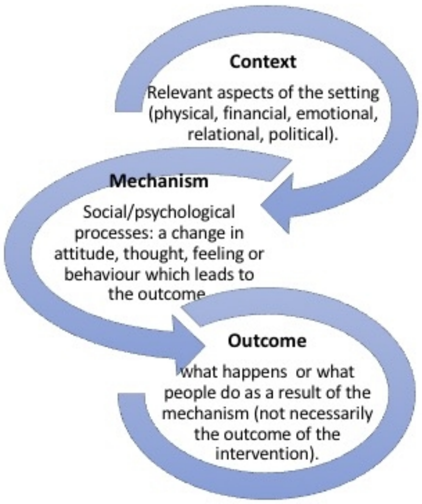

Figure 1_CMO configurations

338x190mm (54 x 54 DPI)

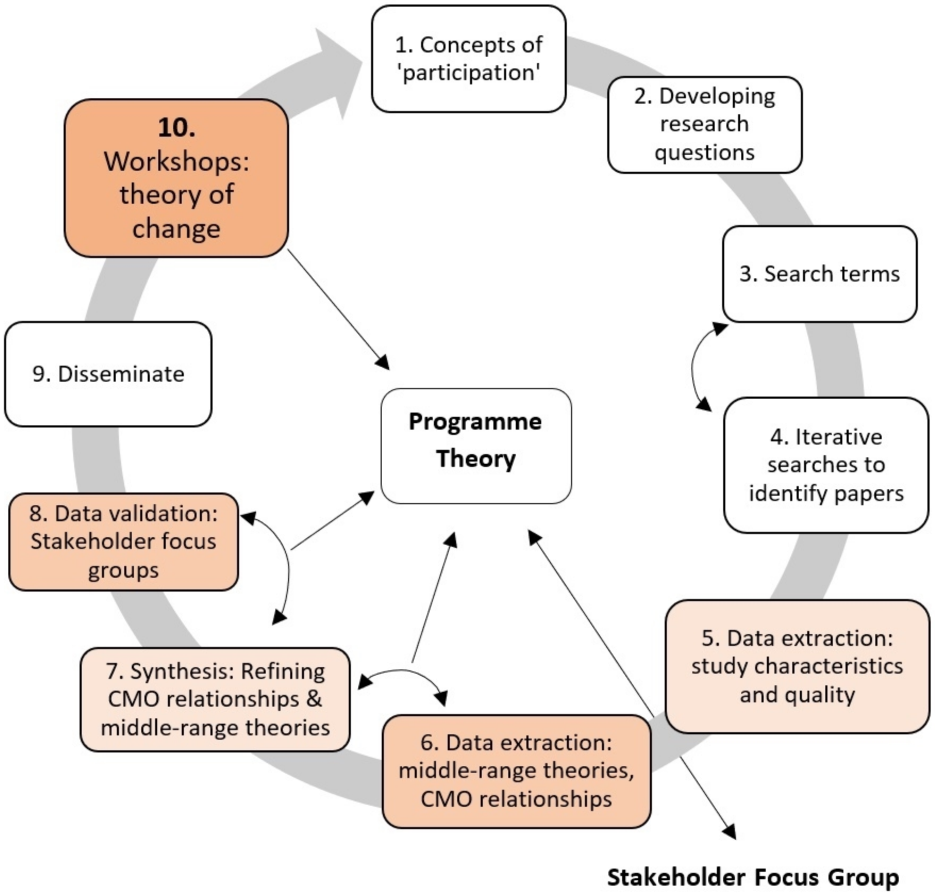

Figure 2: 10 step review process

165x157mm (144 x 144 DPI)

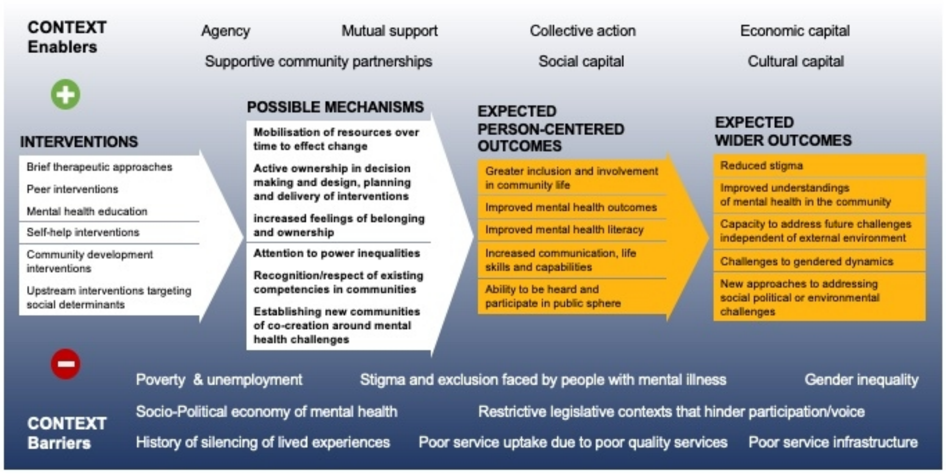

Figure 3: Programme theory

338x190mm (54 x 54 DPI)

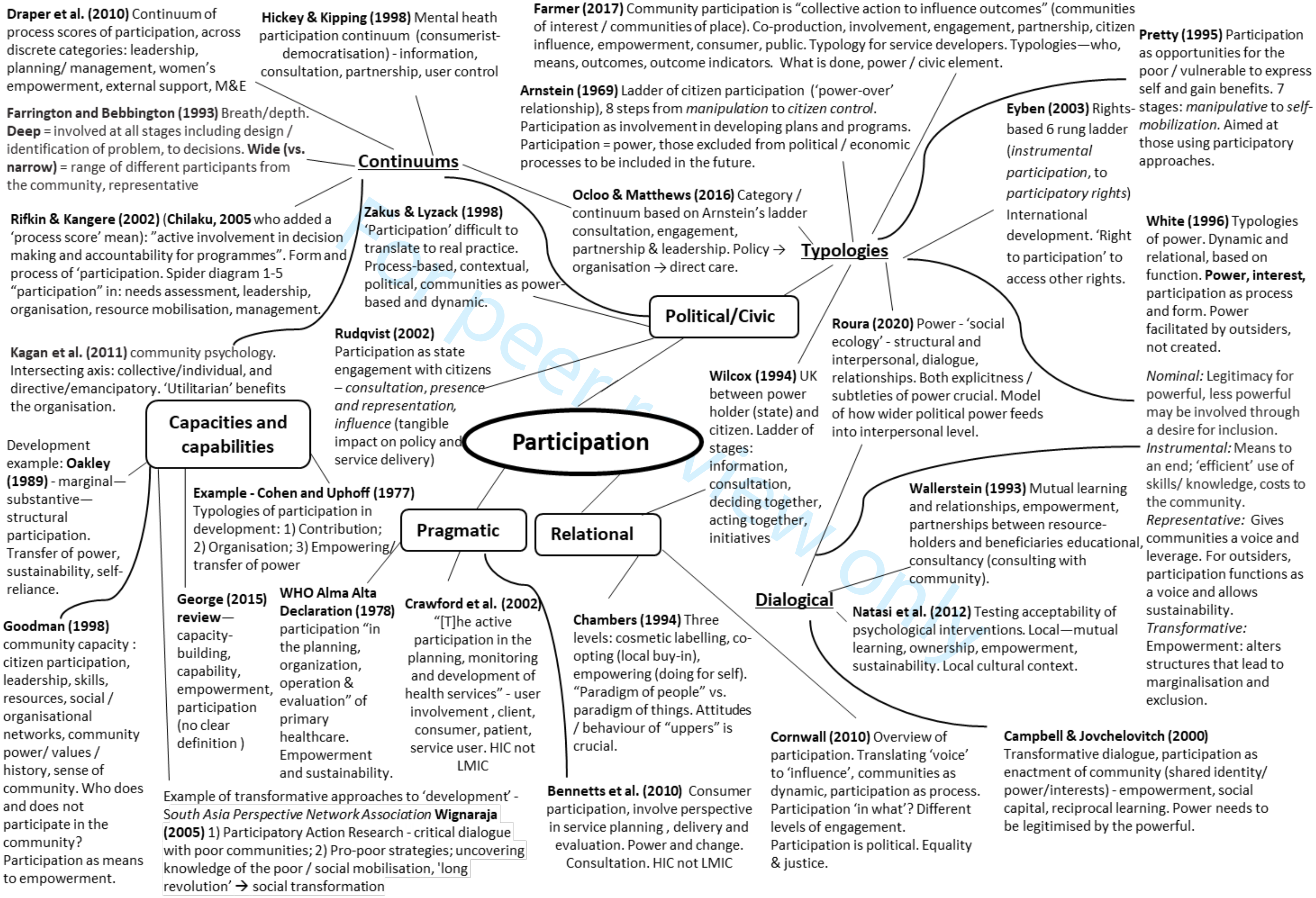
Participation
Continuums
Typologies
Political/Civic
Capacities and capabilities
Pragmatic
Relational
Dialogical
Draper et al. (2010) Continuum of process scores of participation, across discrete categories: leadership, planning/ management, women's empowerment, external support, M&E
Hickey & Kipping (1998) Mental heath participation continuum (consumerist-democratisation) - information, consultation, partnership, user control
Farrington and Bebbington (1993) Breath/depth. Deep = involved at all stages including design / identification of problem, to decisions. Wide (vs. narrow) = range of different participants from the community, representative
Rifkin & Kangere (2002) (Chilaku, 2005 who added a 'process score' mean): "active involvement in decision making and accountability for programmes". Form and process of 'participation. Spider diagram 1-5 "participation" in: needs assessment, leadership, organisation, resource mobilisation, management.
Zakus & Lyzack (1998) 'Participation' difficult to translate to real practice. Process-based, contextual, political, communities as power-based and dynamic.
Kagan et al. (2011) community psychology. Intersecting axis: collective/individual, and directive/emancipatory. 'Utilitarian' benefits the organisation.
Rudqvist (2002) Participation as state engagement with citizens – consultation, presence and representation, influence (tangible impact on policy and service delivery)
Farmer (2017) Community participation is "collective action to influence outcomes" (communities of interest / communities of place). Co-production, involvement, engagement, partnership, citizen influence, empowerment, consumer, public. Typology for service developers. Typologies—who, means, outcomes, outcome indicators. What is done, power / civic element.
Arnstein (1969) Ladder of citizen participation ('power-over' relationship), 8 steps from manipulation to citizen control. Participation as involvement in developing plans and programs. Participation = power, those excluded from political / economic processes to be included in the future.
Ocloo & Matthews (2016) Category / continuum based on Arnstein's ladder consultation, engagement, partnership & leadership. Policy → organisation → direct care.
Eyben (2003) Rights-based 6 rung ladder (instrumental participation, to participatory rights) International development. 'Right to participation' to access other rights.
Pretty (1995) Participation as opportunities for the poor / vulnerable to express self and gain benefits. 7 stages: manipulative to self-mobilization. Aimed at those using participatory approaches.
White (1996) Typologies of power. Dynamic and relational, based on function. Power, interest, participation as process and form. Power facilitated by outsiders, not created.
Nominal: Legitimacy for powerful, less powerful may be involved through a desire for inclusion.
Instrumental: Means to an end; 'efficient' use of skills/ knowledge, costs to the community.
Representative: Gives communities a voice and leverage. For outsiders, participation functions as a voice and allows sustainability.
Transformative: Empowerment: alters structures that lead to marginalisation and exclusion.
Roura (2020) Power - 'social ecology' - structural and interpersonal, dialogue, relationships. Both explicitness / subtleties of power crucial. Model of how wider political power feeds into interpersonal level.
Wilcox (1994) UK between power holder (state) and citizen. Ladder of stages: information, consultation, deciding together, acting together, initiatives
Wallerstein (1993) Mutual learning and relationships, empowerment, partnerships between resource-holders and beneficiaries educational, consultancy (consulting with community).
Natasi et al. (2012) Testing acceptability of psychological interventions. Local—mutual learning, ownership, empowerment, sustainability. Local cultural context.
Development example: Oakley (1989) - marginal—substantive—structural participation. Transfer of power, sustainability, self-reliance.
Example - Cohen and Uphoff (1977) Typologies of participation in development: 1) Contribution; 2) Organisation; 3) Empowering/ transfer of power
Goodman (1998) community capacity : citizen participation, leadership, skills, resources, social / organisational networks, community power/ values / history, sense of community. Who does and does not participate in the community? Participation as means to empowerment.
George (2015) review— capacity-building, capability, empowerment, participation (no clear definition)
WHO Alma Alta Declaration (1978) participation "in the planning, organization, operation & evaluation" of primary healthcare. Empowerment and sustainability.
Crawford et al. (2002) "[T]he active participation in the planning, monitoring and development of health services" - user involvement , client, consumer, patient, service user. HIC not LMIC
Chambers (1994) Three levels: cosmetic labelling, co-opting (local buy-in), empowering (doing for self). "Paradigm of people" vs. paradigm of things. Attitudes / behaviour of "uppers" is crucial.
Example of transformative approaches to 'development' - South Asia Perspective Network Association Wignaraja (2005) 1) Participatory Action Research - critical dialogue with poor communities; 2) Pro-poor strategies; uncovering knowledge of the poor / social mobilisation, 'long revolution' → social transformation
Bennetts et al. (2010) Consumer participation, involve perspective in service planning , delivery and evaluation. Power and change. Consultation. HIC not LMIC
Cornwall (2010) Overview of participation. Translating 'voice' to 'influence', communities as dynamic, participation as process. Participation 'in what'? Different levels of engagement. Participation is political. Equality & justice.
Campbell & Jovchelovitch (2000) Transformative dialogue, participation as enactment of community (shared identity/ power/interests) - empowerment, social capital, reciprocal learning. Power needs to be legitimised by the powerful.

Search strategy for CIHANL, PsycINFO, WebofScience, ASSIA, Medline, Embase

((participat* OR empower* OR community OR "community?led" OR co-designed OR inclusion OR inclusive OR "capacity building" OR capabilities OR engagement OR consultation OR co-produc* OR peer-led OR peer?to?peer OR task?shifting OR task?switching) **AND** ("Psychological disorder" OR "Psychological problem" OR "Psychological illness" OR "Psychological distress" OR "Psychiatric disorder" OR "Psychiatric problem" OR "Psychiatric illness" OR "Psychiatric distress" OR "Psychosocial disability" OR "Mental illness" OR "Mental health" OR SMI) **AND** ("Developing countr*" OR "low?income countr*" OR "middle?income countr*" OR LMIC OR "third world"))

Limits: Peer reviewed

Limits: Date 2007-2021

Search strategy for JSTOR

[JSTOR which has a 200-character word limit. Discussion was had among the team via email and during monthly team meetings, as to how to truncate the search most effectively]

((participat* OR community OR inclus*) AND ab:(psychological OR "psychiatric disorder*" OR "Psychosocial disability*" OR "Mental illness*" OR "Mental health") AND ("developing countries" OR LMIC))

Limits: Date 2007-2021

For peer review only

Reporting checklist for protocol of a systematic review and meta analysis.

Based on the PRISMA-P guidelines.

Instructions to authors

Complete this checklist by entering the page numbers from your manuscript where readers will find each of the items listed below.

Your article may not currently address all the items on the checklist. Please modify your text to include the missing information. If you are certain that an item does not apply, please write "n/a" and provide a short explanation.

Upload your completed checklist as an extra file when you submit to a journal.

In your methods section, say that you used the PRISMA-Preporting guidelines, and cite them as:

Moher D, Shamseer L, Clarke M, Ghersi D, Liberati A, Petticrew M, Shekelle P, Stewart LA. Preferred Reporting Items for Systematic Review and Meta-Analysis Protocols (PRISMA-P) 2015 statement. Syst Rev. 2015;4(1):1.

		Reporting Item	Page Number
**Title**			
Identification	#1a	Identify the report as a protocol of a systematic review	1
Update	#1b	If the protocol is for an update of a previous systematic review, identify as such	N/A
**Registration**			
	#2	If registered, provide the name of the registry (such as PROSPERO) and registration number	16
**Authors**			
Contact	#3a	Provide name, institutional affiliation, e-mail address of all protocol authors; provide physical mailing address of corresponding author	1
Contribution	#3b	Describe contributions of protocol authors and identify the guarantor of the review	19

For peer review only

**Amendments**			
	#4	If the protocol represents an amendment of a previously completed or published protocol, identify as such and list changes; otherwise, state plan for documenting important protocol amendments	N/A
**Support**			
Sources	#5a	Indicate sources of financial or other support for the review	20
Sponsor	#5b	Provide name for the review funder and / or sponsor	20
Role of sponsor or funder	#5c	Describe roles of funder(s), sponsor(s), and / or institution(s), if any, in developing the protocol	20
**Introduction**			
Rationale	#6	Describe the rationale for the review in the context of what is already known	3-8
Objectives	#7	Provide an explicit statement of the question(s) the review will address with reference to participants, interventions, comparators, and outcomes (PICO)	8
**Methods**			
Eligibility criteria	#8	Specify the study characteristics (such as PICO, study design, setting, time frame) and report characteristics (such as years considered, language, publication status) to be used as criteria for eligibility for the review	11
Information sources	#9	Describe all intended information sources (such as electronic databases, contact with study authors, trial registers or other grey literature sources) with planned dates of coverage	10
Search strategy	#10	Present draft of search strategy to be used for at least one electronic database, including planned limits, such that it could be repeated	10-12
Study records - data management	#11a	Describe the mechanism(s) that will be used to manage records and data throughout the review	11
Study records - selection process	#11b	State the process that will be used for selecting studies (such as two independent reviewers) through each phase of the review (that is, screening, eligibility and inclusion in meta-analysis)	11-12
Study records - data	#11c	Describe planned method of extracting data from reports (such as	11-12

For peer review only

collection process		piloting forms, done independently, in duplicate), any processes for obtaining and confirming data from investigators	
Data items	#12	List and define all variables for which data will be sought (such as PICO items, funding sources), any pre-planned data assumptions and simplifications	11-12
Outcomes and prioritization	#13	List and define all outcomes for which data will be sought, including prioritization of main and additional outcomes, with rationale	N/A
Risk of bias in individual studies	#14	Describe anticipated methods for assessing risk of bias of individual studies, including whether this will be done at the outcome or study level, or both; state how this information will be used in data synthesis	13
Data synthesis	#15a	Describe criteria under which study data will be quantitatively synthesised	N/A
Data synthesis	#15b	If data are appropriate for quantitative synthesis, describe planned summary measures, methods of handling data and methods of combining data from studies, including any planned exploration of consistency (such as I2, Kendall's τ)	N/A
Data synthesis	#15c	Describe any proposed additional analyses (such as sensitivity or subgroup analyses, meta-regression)	N/A
Data synthesis	#15d	If quantitative synthesis is not appropriate, describe the type of summary planned	15-16
Meta-bias(es)	#16	Specify any planned assessment of meta-bias(es) (such as publication bias across studies, selective reporting within studies)	N/A
Confidence in cumulative evidence	#17	Describe how the strength of the body of evidence will be assessed (such as GRADE)	N/A

The PRISMA-P elaboration and explanation paper is distributed under the terms of the Creative Commons Attribution License CC-BY. This checklist was completed on 18. September 2021 using https://www.goodreports.org/, a tool made by the EQUATOR Network in collaboration with Penelope.ai

For peer review only
